# Engineered CCR2 positive macrophages coordinate immunoregulation with neural regeneration and matrix remodeling after spinal cord injury

**DOI:** 10.7150/thno.134560

**Published:** 2026-06-10

**Authors:** Yuqi Zhao, Tao Xie, Yanming Ma, Yuhao Wang, Shenghang Liu, Hui Li, Youjun Liu, Renfeng Liu, Hailiang Xu, Cheng Ju, Weidong Wu, Yifan Wang, Siyuan He, Rongjin Luo, Dageng Huang, Shuaijun Jia, Chunping Hu, Liang Yan, Zhiyuan Wang, Lei Zhu

**Affiliations:** 1 Department of Spine Surgery, Honghui Hospital, Xi'an Jiaotong University, Xi'an, Shaanxi 710054, China.; 2 Shaanxi Key Laboratory of Spine Bionic Treatment, Xi'an, Shaanxi 710054, China.

**Keywords:** spinal cord injury, engineered macrophages, mRNA delivery, neural regeneration, multimodal therapy

## Abstract

**Rationale:**

Spinal cord injury (SCI) triggers a complex secondary injury process characterized by inflammation, neuronal loss, extracellular matrix (ECM) disruption, and limited endogenous repair. Although cell-based therapies hold potential for SCI treatment, their efficacy is often constrained by poor lesion targeting, inadequate persistence after delivery, and limited temporal control over therapeutic factor release.

**Methods:**

To address these limitations, we developed a macrophage-based mRNA delivery platform by electroporating CCR2 positive (CCR2^+^) macrophages with ANXA1, GDNF, and CTGF mRNAs. *In vitro*, we assessed transfection efficiency, cell viability, secretion kinetics of therapeutic proteins, anti-inflammatory activity, and neuroprotective and regenerative effects. *In vivo*, using a mouse SCI model, we evaluated lesion-site accumulation, inflammatory regulation, tissue repair, electrophysiological recovery, transcriptomic alterations, and behavioral outcomes.

**Results:**

Following electroporation, CCR2⁺ macrophages efficiently expressed ANXA1, GDNF, and CTGF while maintaining high viability, with no marked shift toward CD86- or CD206-associated phenotypes. These engineered macrophages showed enhanced accumulation at the lesion site and sustained therapeutic protein secretion for up to 14 days. *In vitro*, they protected neuronal cells against oxidative stress-induced injury and promoted neurite outgrowth. *In vivo*, they attenuated inflammation, improved the local repair microenvironment, and promoted axonal regeneration, remyelination, and ECM remodeling, accompanied by partial recovery of electrophysiological and motor function after SCI. RNA-seq analysis further supported broad changes in pathways related to immune regulation, neural repair, myelination, and matrix remodeling.

**Conclusions:**

CCR2-enriched macrophages engineered with reparative mRNAs may represent a promising treatment strategy for SCI. By linking CCR2-associated lesion accumulation with multimodal reparative activity, this cell-based platform provides a potential approach for coordinated microenvironmental regulation and tissue repair.

## Introduction

Spinal cord injury (SCI) remains a major challenge in neurology and rehabilitation medicine because it often results in persistent motor, sensory, and autonomic dysfunction with limited opportunities for meaningful recovery 1, 2. Whether caused by traumatic or non-traumatic insults, SCI imposes a profound physical, psychological, and socioeconomic burden on affected individuals. In the United States alone, approximately 500,000 people are living with SCI, with nearly 11,000 new cases reported each year 3, 4. The lifetime cost of care is estimated to range from $1.1 million to $4.6 million per patient 1. Current treatment still relies largely on early decompression and acute interventions such as high-dose methylprednisolone and mild hypothermia; however, the overall therapeutic benefit of these approaches remains limited. More effective and safer treatment strategies are therefore still urgently needed.

The difficulty of treating SCI is closely related to the complexity of its pathology. The initial mechanical insult is followed by a sustained secondary injury cascade that includes oxidative stress, inflammation, calcium overload, and glutamate excitotoxicity. These pathological events are closely interconnected and progressively amplify tissue damage, ultimately leading to persistent structural loss and neurological dysfunction 5, 6. This multifactorial process may partly explain the limited efficacy of single-target therapies in SCI. More effective treatment is likely to require simultaneous intervention at several levels, including inflammation control, neuroprotection, regenerative support, and extracellular matrix remodeling, in order to reshape the lesion microenvironment and promote repair 7.

Against this background, cell-based therapy has emerged as a potential strategy for SCI repair, and increasing preclinical evidence supports its therapeutic potential 8-10. However, the injured spinal cord is highly heterogeneous and dynamically evolving, which limits the efficacy of unmodified transplanted cells. As a result, engineering transplanted cells to function as active therapeutic carriers is increasingly viewed as an important direction in next-generation cell therapy 11. Compared with conventional drugs or static delivery systems, engineered cells may provide longer *in vivo* persistence, greater responsiveness to local pathological cues, and improved accumulation at the injury site. Related approaches have already shown promise in oncology, where doxorubicin liposome-loaded macrophages have been used to target solid tumors and paclitaxel-loaded neutrophils have been shown to cross the blood-brain barrier in glioma models 12, 13. These findings suggest that similar cell-engineering strategies may also be applicable to neurological disorders, including SCI.

Among candidate cell types, neural stem cells (NSCs), mesenchymal stem cells (MSCs), and olfactory ensheathing cells (OECs) have all been explored for SCI therapy because of their neuroprotective, immunomodulatory, and axon-supportive properties 8, 14, 15. However, these cells are not ideal engineering vehicles 16. Their migration toward the lesion is often weak or nonspecific, which restricts effective enrichment within the injured spinal cord. In addition, genetic or functional modification may compromise cellular stability or performance. NSCs and OECs may show reduced functional consistency after engineering, whereas MSCs, although relatively easy to manipulate, display limited intrinsic tropism toward spinal cord lesions and retain mesodermal differentiation potential. Together with concerns about ectopic distribution, tumorigenic risk, and limited cell availability, these drawbacks highlight the need for a safer and more suitable carrier cell that can be engineered to regulate inflammation, support axonal repair, and remodel the extracellular matrix after SCI 17-19.

Macrophages are particularly attractive in this regard. As key regulators of the post injury inflammatory response, they display substantial phenotypic plasticity and can shift between inflammatory and reparative states in response to local cues 20-22. This property is especially relevant in SCI, where pro-inflammatory macrophages exacerbate tissue damage through mediators such as TNF-α, IL-1β, and iNOS, whereas reparative macrophages help resolve inflammation and support tissue repair by producing IL-10, TGF-β, neurotrophic factors, and extracellular matrix-related components 23. Their functional flexibility makes them well suited for therapeutic programming 24. Macrophages also have a natural tendency to migrate toward injured tissue in response to chemokine gradients 25. Following SCI, increased CCL2 expression at the lesion site promotes recruitment of peripheral CCR2-positive macrophages through the CCL2-CCR2 axis 26-28, providing a biologically relevant basis for lesion-directed homing. In addition, macrophages can be generated in relatively large numbers from peripheral blood mononuclear cells, carry a low tumorigenic risk, and are generally amenable to genetic manipulation. Collectively, these features make macrophages a practical and promising platform for cell-based delivery in SCI.

To utilize these advantages, gene transfer into macrophages must be both efficient and safe. Although viral vectors such as adeno-associated viruses and lentiviruses can achieve high transduction efficiency, their use remains constrained by cost, immunogenicity, and concerns regarding genomic integration 29, 30. mRNA delivery offers a non-integrating alternative that enables rapid yet transient protein expression through direct cytoplasmic translation 31. This feature is particularly relevant in SCI, where early intervention during the secondary injury phase may strongly influence later tissue remodeling and functional recovery. Recent advances in cap structure design, poly(A) tail optimization, and nucleotide modification have further improved the stability and tolerability of therapeutic mRNA 32, 33. Electroporation, in turn, provides a direct and efficient non-viral method for intracellular delivery by transiently permeabilizing the cell membrane, thereby enabling robust expression while minimizing vector-related toxicity and residue 34, 35. Previous studies showing that macrophages loaded with neurotrophic mRNAs can promote repair after SCI further support the feasibility of mRNA electroporation as a macrophage engineering strategy 36-39.

On this basis, we developed a multifunctional macrophage-based therapeutic strategy for SCI by electroporating CCR2-enriched macrophages with ANXA1, GDNF, and CTGF mRNAs. These three cargos were selected to address interconnected aspects of SCI pathology, including dysregulated inflammation, impaired neural repair, and extracellular matrix disruption. ANXA1 was chosen for its pro-resolving and anti-inflammatory actions, including its ability to suppress pro-inflammatory signaling and promote macrophage polarization toward a reparative phenotype 40-42. GDNF was included because of its well-established neurotrophic effects on neuronal survival and axonal growth through GFRα-1-associated signaling pathways 43-46. CTGF was incorporated for its role in extracellular matrix production and remodeling, including the regulation of collagen and laminin, which may contribute to tissue repair 47-49. By combining CCR2-associated homing with multimodal molecular programming, we aimed to establish an engineered macrophage platform capable of coordinated immunomodulation, neurotrophic support, and microenvironmental remodeling for SCI treatment.

In this study, we established an mRNA-based macrophage engineering platform for SCI treatment. CCR2-enriched macrophages were electroporated with ANXA1, GDNF, and CTGF mRNAs to integrate inflammatory regulation, neurotrophic support, and microenvironmental remodeling within a single multimodal therapeutic strategy.

## Materials and Methods

### Isolation of myelin from adult mouse brains

Myelin was isolated from adult C57BL/6 mouse brains using sucrose density-gradient centrifugation, as previously described. Briefly, brains from adult mice were rapidly collected and homogenized in 0.32 M sucrose using a glass–polytetrafluoroethylene homogenizer. The homogenate was carefully layered over 0.85 M sucrose and centrifuged at 27,000 × g for 50 min at 4 °C. Following centrifugation, the crude myelin fraction that settled on top of the interface between 0.32 M and 0.85 M sucrose solutions was obtained. The crude myelin fraction was resuspended in precooled sterile water and subjected to repeated osmotic shock and centrifugation until residual sucrose and visible impurities were removed. The purified myelin was passed through a sterile 0.22 μm filter (Corning) and stored at -80 °C until use.

### Isolation and enrichment of mouse peritoneal macrophages

Peritoneal macrophages were isolated from adult mice using a thioglycollate-elicitation method as previously described 50. Mice received intraperitoneal injections of thioglycollate medium once daily for three consecutive days. On day 4, the peritoneal cavity was lavaged with sterile PBS, and the peritoneal exudate was collected. The lavage fluid was centrifuged at 350 × g for 5 min at 4 °C. The supernatant was discarded, and the cells were resuspended in RPMI-1640 medium containing 5% fetal bovine serum. Cells were incubated at 37 °C for approximately 1 h to allow macrophage adherence. After 1 h of adherence, non-adherent cells were removed by washing three times with precooled PBS, and the remaining adherent macrophages were maintained in culture. The adherent cells were then cultured in fresh RPMI-1640 medium containing 5% FBS for 7 days to allow further maturation. Macrophage purity was confirmed by flow cytometry before subsequent experiments.

CCR2 positive macrophages were further enriched using a modified induction protocol. Briefly, myelin extract (5 μg/mL) was added to thioglycollate medium, and mice received intraperitoneal injections once daily for 3 consecutive days. Peritoneal macrophages were then isolated and cultured as described above. After 7 days of culture, fully differentiated cells were collected for subsequent experiments.

### Flow cytometric sorting of CCR2 positive macrophages

Macrophages were washed 2–3 times with PBS, collected, and centrifuged. The cells were then resuspended in PBS and incubated with anti-CD192 (CCR2) antibody according to the manufacturer’s instructions for 30 min on ice in the dark. After washing to remove unbound antibody, CCR2 positive (CCR2^+^) macrophages were sorted using a NovoCyte flow cytometer (Agilent Technologies).

### Electroporation

Flow-sorted CCR2⁺ macrophages were washed three times with PBS and centrifuged at 500 × g for 5 min before electroporation. For each reaction, 1 × 10^6^ cells were resuspended in electroporation buffer and mixed with 6 μg total mRNA. ANXA1, GDNF, and CTGF mRNAs were combined at a mass ratio of 1:1:1, and 2 μg eGFP mRNA was added as a reporter of transfection efficiency.

The cell–mRNA suspension was transferred to an electroporation cuvette and electroporated using a single 5 ms pulse at 250 V. Immediately after electroporation, cells were transferred to prewarmed complete medium, allowed to recover at room temperature for 10 min, and then returned to standard culture conditions.

At 24 h after electroporation, eGFP fluorescence was examined using an inverted fluorescence microscope (Leica DMi8 THUNDER). Culture supernatants were collected, passed through a sterile 0.22 μm filter membrane (Corning, USA), and stored at -80 °C for subsequent analyses.

### Cell viability after electroporation

Cell viability after electroporation was assessed using a Calcein-AM/PI live/dead assay kit (Beyotime Biotechnology Co., Ltd., China). After staining, cells were observed under an inverted fluorescence microscope. Calcein-AM-positive cells were considered viable cells, whereas PI-positive cells were counted as dead cells. Cell viability was calculated as the proportion of living cells to the total number of cells.

### *In vitro* protein expression kinetics by ELISA

To determine whether macrophages maintained protein secretion after electroporation, culture supernatants were collected on days 1, 3, 5, 7, 10, and 14. After centrifugation to remove cell debris, concentrations of ANXA1, GDNF, and CTGF were measured using ELISA kits according to manufacturers’ instructions.

### Culture and treatment of PC-12 cells and dorsal root ganglia

PC-12 cells were purchased from the Cell Bank of the Chinese Academy of Sciences (Shanghai). PC-12 cells were cultured in RPMI-1640 supplemented with 10% fetal bovine serum (Gibco) and 1% penicillin–streptomycin (100×, Gibco) in a humidified incubator at 37 °C with 5% CO₂.

For subsequent treatment experiments, PC-12 cells were exposed for 24 h to one of the following media: (1) regular culture medium, (2) medium supplemented with 10% unmodified macrophage-conditioned supernatant, or (3) medium supplemented with 10% engineered macrophage-conditioned supernatant.

Newborn Sprague–Dawley rats were first sterilized with 75% ethanol, and the dorsal root ganglia (DRGs) were carefully dissected under sterile conditions using fine forceps. The isolated DRGs were washed in ice-cold D-Hank’s solution, cut into small explants, and cultured in Neurobasal medium supplemented with 1% B27 and 1% penicillin. The explants were cultured in standard dishes under routine conditions. After 7 days *in vitro*, DRG explants were stained with the neuronal marker Tuj1. Images from random fields were acquired using an inverted fluorescence microscope (DMi8 Thunder, Leica). Neurite outgrowth was quantified with ImageJ, using the average length of the 50 longest neurites and the total neurite area as evaluation parameters 51, 52.

To model oxidative stress, PC-12 cells and DRG explants were exposed for 3 h to one of the following treatments: (1) 200 μM H₂O₂ alone, (2) 200 μM H₂O₂ plus 10% macrophage-conditioned supernatant, or (3) 200 μM H₂O₂ plus 10% engineered macrophage-conditioned supernatant.

### Apoptosis assay

To assess the effects of engineered macrophages on PC-12 cell apoptosis under both basal and oxidative stress conditions, cells were cultured in the corresponding treatment media and then analyzed by TUNEL staining using a one-step apoptosis detection kit (red fluorescence, AF594, E-CK-A322, Elabscience, China). After treatment, cells were fixed with 4% paraformaldehyde, permeabilized with 0.3% Triton X-100 for 15 min. The samples were then incubated at 37 °C for 30 min, followed by an additional 60 min reaction in the dark. After that, the nuclei were stained with DAPI. Fluorescence images were captured using a THUNDER Imager Tissue microscope (Leica). Apoptotic cells were quantified with ImageJ software, and the apoptotic index was calculated as the percentage of TUNEL-positive cells among the total number of cells. Image acquisition and quantitative analysis were performed in a blinded manner with respect to the experimental groups.

### Live and dead cell assay

To assess the effects of engineered macrophages on PC-12 cell survival under both basal and oxidative stress conditions, cells were cultured in the corresponding conditioned media according to the experimental grouping described above. Cell viability and cytotoxicity were determined using a Calcein-AM/PI Cell Viability and Cytotoxicity Assay Kit (C2015M, Beyotime, China) in accordance with the manufacturer’s protocol. After staining, images from random fields were captured using an inverted fluorescence microscope (DMi8 Thunder, Leica). Cell viability was calculated as the percentage of Calcein-AM-positive cells among the total number of Calcein-AM-positive and PI-positive cells, using the following formula: Cell viability (%) = Number of Calcein-AM⁺ cells / (Number of Calcein-AM⁺ cells + Number of PI⁺ cells) × 100%. In addition, the proportions of live (Calcein-AM⁺) and dead (PI⁺) cells relative to the total cell number were also quantified to reflect cell survival and cytotoxicity.

### Cell proliferation assay

To detect the impact of engineered macrophages on the proliferation of PC-12 cells, the cells were inoculated into appropriate conditioned media according to the grouping design. Cell proliferation was evaluated using a BeyoClick™ EdU cell proliferation kit (Alexa Fluor 488, C0071S, Beyotime, China). Briefly, cells were fixed with 4% paraformaldehyde, permeabilized with 0.2% Triton X-100, and incubated with the EdU reaction mixture at room temperature for 30 min. Images were acquired using a THUNDER Imager microscope, and EdU-positive cells were quantified using ImageJ software. Proliferation is expressed as the percentage of EdU-positive cells to all cells.

### Western blot analysis

Western blot analysis was carried out using conventional procedures. After 3 days of culture, macrophages and engineered macrophages were collected, and total protein was extracted with RIPA lysis buffer (Beyotime, China). Protein concentration was measured using a BCA Protein Assay Kit (Beyotime, China). Equal amounts of protein were then separated by 12% SDS-PAGE and transferred onto PVDF membranes (Millipore, USA). The membranes were blocked with 5% non-fat milk and incubated overnight with primary antibodies against GDNF (1:1000, mouse monoclonal, Santa Cruz), ANXA1 (1:1000, mouse monoclonal, Santa Cruz), CTGF (1:1000, mouse monoclonal, Santa Cruz), and β-tubulin (1:1000, ab52866, Abcam), which was used as the loading control. After washing five times with PBST, the membranes were incubated with an HRP-conjugated secondary antibody (1:1000, A0208, Beyotime) at room temperature in 5% non-fat milk. Protein bands were visualized using ECL chemiluminescent substrate (Millipore), and images were acquired with a ChemiDoc imaging system (Bio-Rad, USA).

### Spinal cord injury model and intravenous administration of macrophages

Female C57BL/6 mice (8 weeks old, 18–22 g; n = 98) were obtained from the Laboratory Animal Center of Xi’an Jiaotong University. All animal procedures were approved by the Animal Ethics Committee of Honghui Hospital, Xi’an Jiaotong University (Approval No. XJTUAE2025-2117), and were performed in accordance with institutional guidelines and the NIH Guide for the Care and Use of Laboratory Animals.

Mice were anesthetized with 1% sodium pentobarbital (50 mg/kg, intraperitoneally). After exposure of the T8 spinal cord, a vascular clip was applied for 30 s to establish a contusive SCI. Mice were randomly allocated to three experimental groups: engineered macrophage group, macrophage group, and SCI control group. For long-term (8-week) studies, including histology, tracer injection, biodistribution and allowance for experimental loss, 24, 10, and 10 mice were allocated to the three groups, respectively. Additional mice were allocated as follows: day 3 histology, 3 mice per group (n = 9); day 7 histology, 3 mice per group (n = 9); day 7 flow cytometry, 3 mice per group (n = 9); RNA-seq, 3 mice per group (n = 9); electrophysiology, 4 mice per group (n = 12); sham controls, 6 mice.

For intravenous cell administration, unmodified macrophages and engineered macrophages were collected, washed with sterile PBS, and resuspended in sterile saline at a concentration corresponding to 1 × 10^6^ cells per mouse. Before administration, both unmodified and engineered macrophages were labeled with DiR by incubation with 1 μL DiR dye (1-octadecyl-3,3,3,3-tetramethylindotricarbocyanine iodide, 100 μg/mL; Aladdin, China) in 200 μL sterile saline. After labeling, the cells were washed to remove unbound dye and resuspended in sterile saline for injection. A total volume of 10 μL of the cell suspension was slowly infused through the tail vein using a microinjection pump at a constant rate of 1 μL/min for 10 min. Mice in the SCI control group received an equal volume of sterile saline through the tail vein using the same infusion procedure. After administration, the injection site was gently compressed until bleeding ceased, and the mice were returned to their cages for postoperative care.

### Flow cytometry analysis

For tissue flow cytometry, a 3 mm spinal cord segment centered on the lesion was collected 7 days after the injury. The tissue was chopped and mechanically separated by a 70 μm cell sieve to prepare a single cell suspension. Cells were centrifuged at 300 × g for 10 min at 4 °C, resuspended, and incubated with the indicated antibodies for 30 min at 4 °C. After staining, the samples were fixed with 1% paraformaldehyde before flow cytometric acquisition.

The following antibodies were used: anti-mouse CD11b-APC (1:200, 562102, BD Pharmingen), anti-mouse CD45-PE (1:200, 157604, BioLegend), anti-mouse CD86-FITC (1:200, 561961, BD Pharmingen), and anti-mouse CD206-MMR (1:200, 321126, BioLegend). Samples were analyzed on a NovoCyte flow cytometer (Agilent), and the data were processed using FlowJo v7.6 software.

### Inflammatory cytokine quantification

In order to evaluate the inflammatory response after injury, a 3 mm spinal cord segment centered on the lesion site was collected on the 7th day after the injury. Each sample was weighed and homogenized in cold PBS containing protease inhibitors, and the final homogenate volume was adjusted according to tissue mass. The homogenate was passed through a 70 μm cell strainer and further disrupted by sonication.

Concentrations of IL-1β, IL-4, IL-6, IL-10, and TNF-α were measured using ELISA kits (Qingdao Ruikai Biotechnology Co., Ltd) according to the manufacturer’s instructions. Absorbance was read using a microplate reader, and cytokine concentrations were calculated from standard curves.

### *In vivo* imaging and axonal tracing

After tail-vein injection of macrophages, the AniView600 small animal imaging system (BLT, China) was used for imaging at 30 min, 48 h and 7 days respectively. The excitation and emission wavelengths were 748 nm and 780 nm, respectively. On the seventh day, mice were deeply anesthetized and perfused; the spinal cord, lungs, kidneys, liver, spleen, and heart were collected for *in vitro* imaging under the same collection conditions. Fluorescence intensity was quantified to assess the biodistribution and lesion-associated accumulation of the transplanted cells. Anterograde and retrograde axonal tracing experiments were performed 1 week prior to tissue collection. Mice were anesthetized with 1% sodium pentobarbital (40 mg/kg, intraperitoneally) and fixed in a stereotaxic apparatus. Four burr holes (~1 mm diameter) were drilled in the bilateral motor cortex (coordinates in mm: AP 1.0/1.0, AP 1.0/–1.0, AP –1.0/1.0, AP –1.0/–1.0; depth: 0.5 mm). A microsyringe was mounted vertically on the stereotaxic injector arm, and 0.5 μL of 10% biotinylated dextran amine (BDA, Thermo Fisher, USA) was injected into each site for anterograde tracing. In the same animals, retrograde tracing of sensory pathways was performed by injecting 1.2 μL of 2% Fast Blue (FB) into four bilateral sites located 5–6 mm caudal to the injury site (approximately at the L1/L2 spinal level), at a depth of 0.5 mm. After injection, the surgical site was sutured, and mice received intramuscular penicillin for 3 consecutive days to prevent infection. One week after tracer injection, mice were anesthetized and perfused. Spinal cord tissues were collected and processed for cryosectioning and subsequent tracing analysis.

### Spinal cord electrophysiology

At 8 weeks after spinal cord injury, motor evoked potentials (MEPs) were recorded to evaluate the recovery of conduction-related function. Mice were anesthetized with 1% sodium pentobarbital (50 mg/kg, intraperitoneal injection) and fixed in a stereotaxic frame. Recordings were obtained with the MadLab-4C data acquisition and analysis system (Zhongshi Technology, China). For stimulation, a pair of tungsten microelectrodes (85 mm, 0.5 MΩ, KeDouBC) was used as bipolar electrodes. The two electrodes were placed 1 mm apart and inserted into the T6 spinal segment at a depth of about 300–500 μm. Single square-wave pulses with a duration of 0.1 ms were delivered every 3 s. For recording, a 1 mm silver ball electrode was placed at the T10 segment. The signals were filtered with a 20–1000 Hz bandpass and then analyzed with MATLAB software. Parameters such as amplitude and latency were quantified in a blinded manner by technicians who were unaware of the treatment groups.

### Electromyography (EMG) recording

At 8 weeks post-spinal cord injury, electrode implantation was performed to record electromyography (EMG) signals from the hindlimbs, in order to assess the recovery of muscle motor function. A non-invasive chip electrode was used, consisting of two stimulation electrodes and one recording electrode. In a quiet and enclosed environment, the chip electrodes were attached to the surface of the gastrocnemius and tibialis anterior muscles of awake mice. During spontaneous movement, EMG signals were continuously acquired and transmitted by a bioelectrical signal acquisition system. The electrical activity of the extensor and flexor muscles in the right hindlimb was recorded during free movement, and synchronized with a digital oscilloscope. Joint movement was observed at the same time to identify the stance and swing phases during locomotion. The recorded EMG signals were filtered with a 20–1000 Hz bandpass and then analyzed using MATLAB. All recordings and data analysis were performed in a blinded manner, with investigators unaware of the group allocation. Muscle activity was evaluated based on the amplitude and rhythmic pattern of the EMG signals.

### Muscle atrophy analysis

Eight weeks after the operation, the bilateral gastrocnemius muscle was removed from each experimental group (n = 6) and weighed. In order to provide normal reference values, we also collected bilateral gastrocnemius muscles of age-matched female C57BL/6 mice (8 weeks old, weight 18–22 g) and compared their average wet weight.

Muscle samples were fixed in 4% paraformaldehyde for 72 h, dehydrated, embedded in paraffin, and sectioned at 5 μm. Sections were stained with H&E and Masson’s trichrome. Bright-field images were acquired at 200× magnification using a Leica DMi8 microscope. The cross-sectional area of muscle fibers and the collagen-positive area were quantified using ImageJ.

### Behavioral assessment

Motor recovery was assessed weekly for 8 weeks using the Basso Mouse Scale (BMS). Before scoring, each mouse was placed in an open field for acclimation. Hindlimb locomotor function was then evaluated according to the standard BMS criteria by investigators blinded to group allocation.

In order to further study the movement function, the GREEN Walk system and its supporting software were used for automatic gait analysis on the day before tissue collection. The system consists of a narrow glass walkway, which is illuminated by green LED lights above the walkway, and a high-speed camera is placed below to capture the position of the paw and gait parameters. The mice were trained continuously for 3 days before the official test. During the test, each mouse completed three runs on the walkway. The recorded parameters include stride, step time, step frequency, movement speed, swing time and maximum paw contact area.

### Perfusion sectioning and immunofluorescence staining

PC-12 cells were seeded onto glass coverslips and cultured to the desired density. Cells were fixed with 4% paraformaldehyde for 15 min and permeabilized with 0.3% Triton X-100 in PBS for 10 min. After blocking with BSA for 25 min at room temperature, the cells were incubated with Tuj1 (1:500, Abcam), NF (1:500, Abcam) and MAP2 (1:200, Santa Cruz) antibodies at 4 °C overnight. The next day, cells were washed three times with PBS, and then incubated at room temperature with the corresponding Alexa Fluor 488, 555, 594 or 647-labeled secondary antibodies (1:200, Invitrogen) for 2 h. Nuclei were counterstained with DAPI. Slides were mounted with anti-fade mounting medium and images were acquired using a DMi8 THUNDER microscope (Leica). Images were processed and analyzed using Leica LAS X 3.5.1 and ImageJ.

Mice were deeply anesthetized with 1% sodium pentobarbital (40 mg/kg), perfused with cold heparinized saline, and then perfused with 4% paraformaldehyde (PFA). The spinal cord was then carefully removed, post-fixed in 4% PFA at 4 °C for 48 h, dehydrated in sucrose, embedded, and cryosectioned into 16 μm thick sections. Sections were obtained from a 6–8 mm spinal cord segment centered on the lesion site. Sections were washed with PBS, permeabilized with 0.1% Triton X-100, blocked with 5% BSA, and incubated overnight with the indicated primary antibodies at 4 °C. Primary antibodies against Tuj1 (1:500; Abcam), GFAP (1:200; Abcam), Syn (1:200; Santa Cruz), NF (1:300; Abcam), MBP (1:300; Proteintech), FN (1:300; Abcam), LN (1:300; Abcam), CCL2 (1:200; Santa Cruz), IBA-1 (1:200; Abcam), IL-10 (1:300; Proteintech), IL-1β (1:300; Proteintech), Caspase-3 (1:200; Proteintech), MPO (1:200; Proteintech), NeuN (1:200; Proteintech), GDNF (1:200; Santa Cruz), GFRα-1 (1:200; Santa Cruz), NG2 (1:300; Proteintech), Brevican (1:200; Proteintech), ANXA1 (1:200; Santa Cruz), and FPR2 (1:200; Santa Cruz) were used at the dilutions listed above. After PBS washing, sections were incubated with Alexa Fluor-conjugated secondary antibodies (1:200, Invitrogen) at room temperature for 2 h away from light. Nuclei were counterstained with DAPI (1:1000, Beyotime). Images were acquired using a DMi8 THUNDER wide-field fluorescence microscope. Images were processed and analyzed using Leica LAS X 3.5.1 and ImageJ.

### Morphological analysis of IBA-1 positive inflammatory cells

To further evaluate the activation status of IBA-1⁺ inflammatory cells, we carried out a morphological analysis using ImageJ. All images were collected from peri-lesional areas under the same exposure conditions. For each animal, 3–5 random fields were selected, and 5–10 IBA-1⁺ cells were randomly chosen from each field for measurement. The cell body area and circularity were determined after manually outlining the soma. To analyze cellular processes, the images were first binarized and then skeletonized, and the total process length was measured using the Analyze Skeleton plugin. All measurements were performed in a blinded manner.

### Transmission electron microscopy

For ultrastructural analysis, the mice were deeply anesthetized with 1% pentobarbital sodium (40 mg/kg), perfused with cold heparinized saline (10 U/mL) through the heart, and then perfused with 50 mL of 4% glutaraldehyde. Spinal cord tissue containing the lesion site was collected and further fixed in 4% glutaraldehyde at 4 °C for 3 days. Samples were dehydrated, embedded, and sectioned into ultrathin sections of approximately 60 nm. After staining with 5% uranyl acetate and 0.3% lead citrate, sections were examined using an H-600 transmission electron microscope (Hitachi, Japan). The area of regenerated axons and the proportion of myelinated axons in each field were quantified. The degree of myelination was further evaluated by calculating the g ratio, which is defined as the inner diameter of the axon divided by the outer diameter of the fiber.

### RNA extraction and analysis

The spinal cord tissue was collected for transcription analysis 3 days and 8 weeks after the injury respectively. Total RNA was extracted using TRIzol reagent (Invitrogen). RNA quality was assessed using an Agilent 5300 Bioanalyzer, and RNA concentration was measured using an ND-2000 spectrophotometer. RNA purification, reverse transcription, library construction and sequencing were all completed by Shanghai Biotechnology Co., Ltd. (Shanghai, China).

For library construction, 1 μg total RNA from each sample was processed using the Illumina Stranded mRNA Prep, Ligation kit (Illumina, San Diego, CA, USA). Libraries were quantified using a Qubit 4.0 fluorometer and sequenced on a NovaSeq 6000 platform with 2 × 150 bp paired-end reads. Raw reads were trimmed and quality-controlled using fastp. High-quality reads were aligned to the reference genome using HISAT2, and transcripts were assembled based on reference annotations using StringTie. To further analyze changes in cellular composition within the spinal cord tissue, transcriptomic data were subjected to deconvolution analysis using BayesPrism, with reference single-cell RNA-seq datasets obtained from previously published studies 53, 54.

### Quantitative real time PCR analysis

To evaluate *Ccl2* expression at 3 days after injury and the expression of SCI-related genes at 8 weeks, qPCR was performed on tissues collected from the lesion area. Samples were collected at the indicated time points and immediately frozen. Total RNA was extracted, reverse-transcribed into cDNA, and subjected to qPCR using SYBR Green Master Mix. Relative gene expression was calculated using the 2^-ΔΔCt^ method and normalized to *Gapdh*. Primer sequences are listed in Table S1.

### Statistical analysis

All statistical analyses were performed using SPSS and GraphPad Prism 9.0 software. Data are presented as mean ± standard deviation (SD). Differences among multiple groups were analyzed using one-way analysis of variance (ANOVA) followed by Tukey’s post hoc test. Comparisons between two groups were performed using an unpaired two-tailed Student’s t-test. Longitudinal BMS scores were analyzed using repeated-measures two-way ANOVA, with treatment group and time as the two factors, followed by Tukey’s post hoc test for multiple comparisons. A value of p < 0.05 was considered statistically significant.

## Results and Discussion

### Chemokine upregulation after SCI and generation of CCR2 positive engineered macrophages

**Figure 1:**
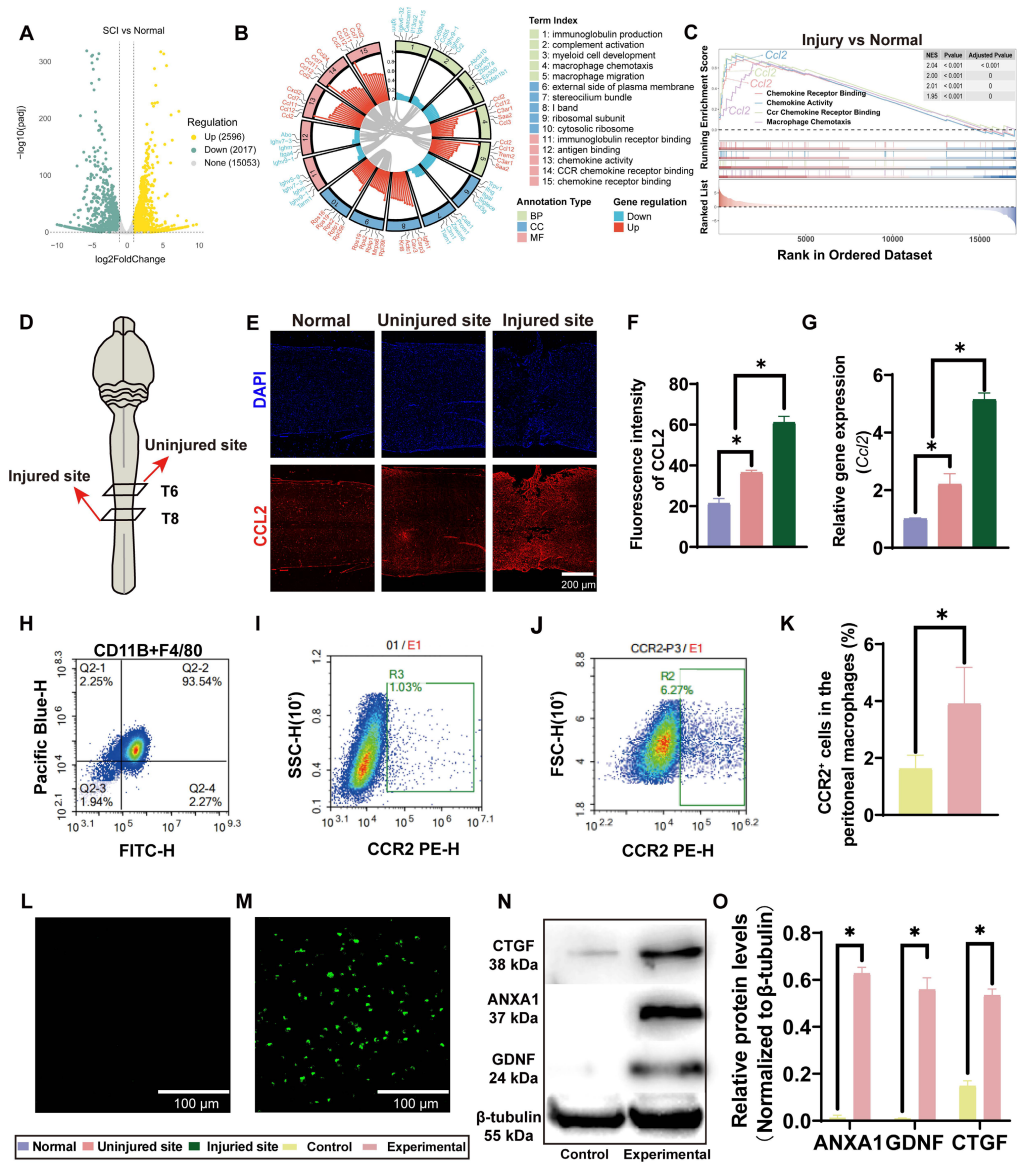
** Activation of CCL2/CCR2 axis and formation of engineered CCR2+ macrophages in SCI.** (A) Volcano plot of the differential expression of genes in the injury group compared with the normal group. The yellow, green and gray dots represent upregulated genes, downregulated genes and genes without significant changes respectively. (B) GO enrichment analysis of differentially expressed genes. The circular diagram shows the enriched biological process (BP), cellular component (CC) and molecular function (MF) entries, among which the chemokine-related pathways (such as chemokine activity, chemokine receptor binding and macrophage chemotaxis) are significantly enriched. (C) Gene set enrichment analysis showing enrichment of Ccl2-associated chemotactic signatures in injured spinal cord tissue. (D) Schematic of tissue sampling from uninjured cords and cords at day 3 post-SCI. (E) Representative immunofluorescence images showing CCL2 staining (red) and DAPI-labeled nuclei (blue) in spinal cord sections from day-3 SCI and control mice (Scale bar: 200 μm). (F) Quantitative analysis of CCL2 fluorescence intensity. (G) Differential expression of *Ccl2* mRNA. (H) Flow-cytometric gating of CD11b⁺/F4/80⁺ peritoneal macrophages. (I-J) CCR2 expression in peritoneal macrophages before and after myelin-extract stimulation. (K) Quantitative analysis of CCR2 expression in peritoneal macrophages before and after stimulation with myelin extract. (L–M) eGFP expression in peritoneal macrophages before and after electroporation (scale bar, 100 μm). (N) Western blot of ANXA1, CTGF, and GDNF in macrophages before and after electroporation. (O) Densitometric quantification of Western blots for ANXA1, CTGF, and GDNF expression in peritoneal macrophages before and after electroporation. Data are presented as mean ± SD; n = 3 biologically independent samples per group. Statistical significance was determined by unpaired two-tailed Student’s t-test or one-way ANOVA, as appropriate. *p < 0.05.

To characterize early transcriptional changes within the lesion core after SCI, we performed RNA sequencing on spinal cord tissues collected from normal mice and from the lesion epicenter at 3 days post-injury. Based on the criteria of |log2FC| > 1.5 and p < 0.05, a total of 4613 differentially expressed genes (DEGs) were identified, including 2596 upregulated and 2017 downregulated genes (Figure 1A). Gene Ontology (GO) enrichment analysis showed that CCL2-related signaling was prominently represented among the altered pathways, including macrophage chemotaxis, chemokine receptor binding, and chemokine activity (Figure 1B). Consistently, Gene Set Enrichment Analysis (GSEA) revealed significant enrichment of CCL2-associated chemokine signaling and macrophage recruitment signatures in injured spinal cord tissue compared with normal tissue (enrichment score = 2.04, p < 0.001, adjusted p < 0.001; Figure 1C). Consistent with these findings, CCL2 expression was elevated in the injured spinal cord. The mean fluorescence intensity of CCL2 in normal, uninjured, and injured sites was 21.37 ± 2.41, 36.50 ± 1.12, and 61.10 ± 3.00, respectively, indicating a 2.86-fold increase at the injury site compared to normal tissue, and a 1.67-fold increase compared to the adjacent uninjured segment (Figure 1D-F). These findings were further validated by qPCR, confirming the significant upregulation of *Ccl2* in the injured tissue (Figure 1G).

Because CCL2-related chemotactic signaling was activated early after SCI, we focused on CCR2, a major receptor for CCL2, as a practical marker for enriching macrophages with lesion-associated accumulation potential. Flow cytometric analysis showed that more than 93% of the isolated peritoneal cells were CD11b^+^&F4/80^+^, indicating that the cell preparation consisted predominantly of macrophages (Figure 1H). However, the baseline proportion of CCR2^+^ cells remained low, accounting for only approximately 1.62% of the population (Figure 1I). To increase this fraction, mice were pretreated with intraperitoneal myelin extract before macrophage isolation. This increased the proportion of CCR2^+^ macrophages to approximately 3.90% (Figure 1J), corresponding to an approximately 2.4-fold increase over the untreated group (Figure 1K). Although the absolute increase was modest, the effect was consistent and statistically significant, suggesting that myelin extract enhances CCR2 expression in macrophages.

Because unmodified macrophages expressed only low levels of ANXA1, CTGF, and GDNF, we introduced mRNAs encoding these three proteins into CCR2^+^ macrophages by electroporation. eGFP mRNA was included as a reporter of transfection efficiency. After electroporation, clear green fluorescence was readily detected in macrophages, confirming successful expression of the introduced eGFP mRNA (Figure 1L, M). Western blot analysis further demonstrated marked upregulation of ANXA1, CTGF, and GDNF proteins, which were nearly undetectable in unmodified macrophages (Figure 1N, O). Together, these data indicate that the exogenous mRNAs were successfully delivered into macrophages and efficiently translated into the intended therapeutic proteins.

We next examined whether electroporation itself altered macrophage polarization. Flow cytometric analysis of CD11b, CD86, and CD206 showed that electroporation did not markedly increase either CD86⁺ or CD206⁺ macrophage subsets. Specifically, 96.47% of cells were distributed in the CD11b^+^CD86^-^ quadrant, whereas only 1.32% were CD11b^+^CD86^+^. Similarly, 98.89% of cells were CD11b^+^CD206^-^, whereas only 1.07% were CD11b^+^CD206^+^ (Figure S1). These findings suggest that electroporation did not cause a detectable shift toward either a CD86-associated inflammatory phenotype or a CD206-associated reparative phenotype under the tested conditions.

The feasibility of this strategy was further supported by viability and secretion analyses. Live/dead staining showed that most macrophages remained viable after mRNA delivery, with no significant reduction in survival compared with untreated controls (Figure S2A, B). ELISA of culture supernatants collected on days 1, 3, 5, 7, 10, and 14 showed sustained release of ANXA1, GDNF, and CTGF throughout the observation period (Figure S2C-E). Secretion of all three proteins peaked around days 5-7 and remained detectable on day 14. These findings indicate that electroporated macrophages can provide sustained but time-limited therapeutic protein expression during a period that substantially overlaps with the early critical phase of secondary SCI pathology.

Taken together, these results show that early CCL2-related chemotactic signaling after SCI can be exploited to enrich and engineer CCR2^+^ macrophages as a multifunctional delivery platform. The resulting cells retained high viability, showed no marked CD86/CD206-defined phenotypic shift under the tested conditions, and supported sustained short-term expression of multiple therapeutic proteins, supporting their further evaluation as a therapeutic strategy for SCI.

These findings provide the cellular and mechanistic basis for the engineered macrophage strategy used in this study. After SCI, the CCL2–CCR2 axis represents a biologically relevant recruitment pathway for peripheral macrophages, and may therefore be exploited to improve lesion-associated cell accumulation 26-28. In parallel, mRNA electroporation provides a transient and non-integrating approach for therapeutic protein expression, reducing concerns related to viral vector integration and long-term uncontrolled expression 29-36. By combining CCR2-associated lesion homing with programmable expression of ANXA1, GDNF, and CTGF, this platform was designed to address inflammation, neural repair, and extracellular matrix remodeling within a single therapeutic system.

### Neuroprotective and neurite outgrowth promoting effects of engineered macrophages *in vitro*

**Figure 2:**
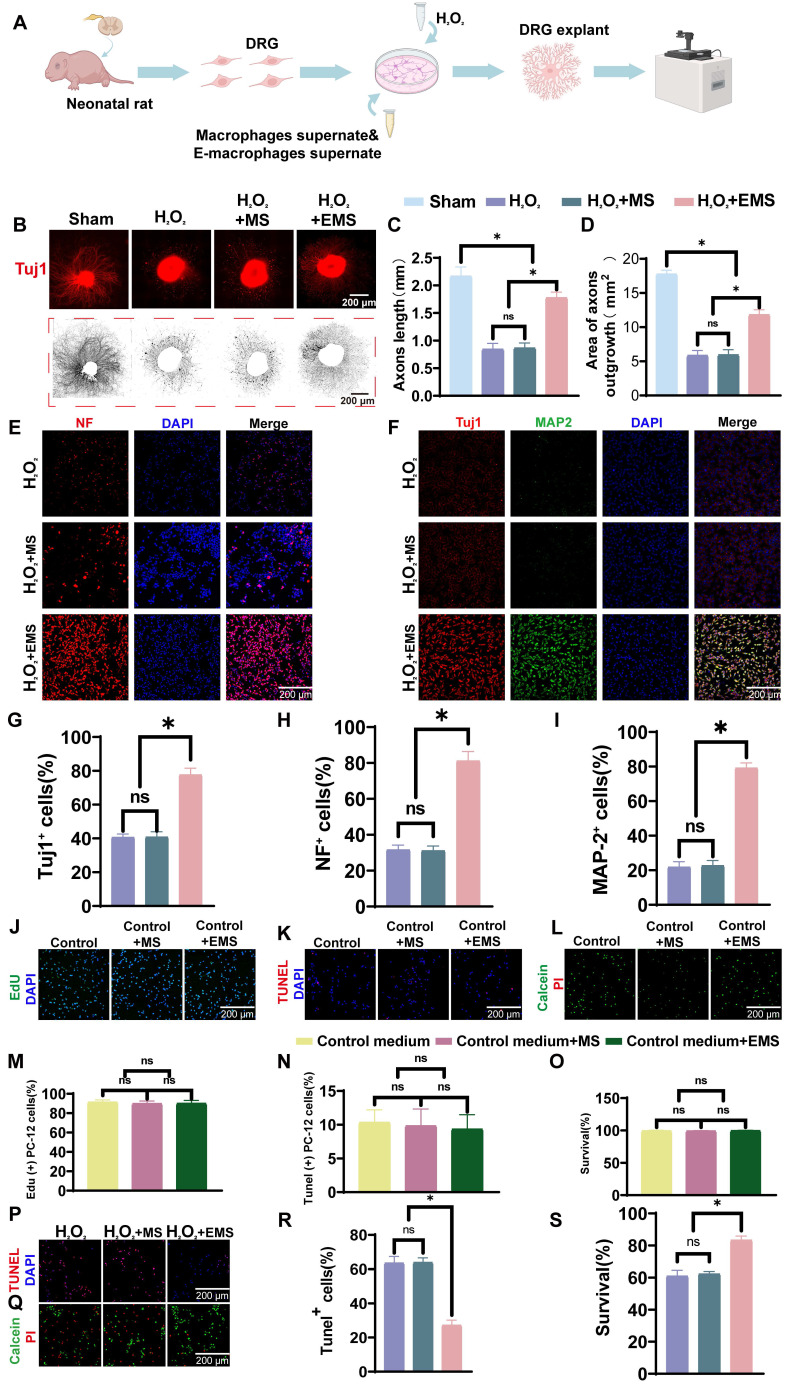
**Neuroprotective and neurite/axonal growth-promoting effects of engineered macrophages *in vitro*.** (A) Schematic overview of the extraction, culture, treatment, and evaluation of DRG explants. (B) Representative Tuj1 staining images of DRG explants and corresponding NeuriteJ-processed images under sham, H₂O₂, H₂O₂ + macrophage supernatant (MS), and H₂O₂ + engineered macrophage supernatant (EMS) conditions. (Scale bar: 200 μm) (C) Quantification of axon length in DRG explants. (D) Quantification of axonal outgrowth area in DRG explants. (E) Representative immunofluorescence staining of NF (red) with DAPI nuclear counterstaining (blue) in PC-12 cells exposed to oxidative stress. (Scale bar: 200 μm) (F) Representative immunofluorescence staining of Tuj1 (red), MAP2 (green), and DAPI (blue) in PC-12 cells under oxidative stress. (Scale bar: 200 μm) (G–I) Quantification of the percentages of Tuj1⁺, NF⁺, and MAP2⁺ cells, respectively. (J) Representative EdU staining images of PC-12 cells cultured under basal conditions. (Scale bar: 200 μm) (K) Representative TUNEL staining images of PC-12 cells cultured under basal conditions. (Scale bar: 200 μm) (L) Representative Calcein-AM/PI staining images of PC-12 cells cultured under basal conditions. (Scale bar: 200 μm) (M–O) Quantification of EdU⁺ proliferating cells, TUNEL⁺ apoptotic cells, and cell survival under basal conditions, respectively. (P) Representative TUNEL staining images of PC-12 cells under H₂O₂-induced oxidative stress. (Scale bar: 200 μm) (Q) Representative Calcein-AM/PI staining images of PC-12 cells under H₂O₂-induced oxidative stress. (Scale bar: 200 μm) (R–S) Quantification of TUNEL⁺ apoptotic cells and cell survival under H₂O₂-induced oxidative stress. Scale bars are indicated in the corresponding panels. Data are presented as mean ± SD; n = 3 biologically independent experiments per group. Statistical significance was determined by one-way ANOVA. *p < 0.05; ns, not significant.

To examine the *in vitro* activity of engineered macrophages, we established a conditioned-medium workflow using both PC-12 cells and dorsal root ganglion (DRG) explants (Figure 2A). In this system, neuronal cells or DRG explants were exposed to conditioned medium collected from unmodified or engineered macrophages, with or without H₂O₂ challenge, to evaluate neuroprotection and neurite/axonal growth-related effects.

We first assessed whether engineered macrophage-conditioned medium could promote neurite/axonal outgrowth in DRG explants. Representative Tuj1 staining together with NeuriteJ analysis showed that H₂O₂ markedly impaired neurite extension, whereas treatment with engineered macrophage-conditioned medium substantially improved radial neurite outgrowth (Figure 2B). Quantitative analysis showed that the average axon length in the sham group was 2.17 ± 0.17 mm, but decreased to 0.84 ± 0.11 mm and 0.87 ± 0.09 mm in the H₂O₂ group and the H₂O₂ + macrophage supernatant group, respectively (Figure 2C). In contrast, axon length increased to 1.78 ± 0.10 mm in the H₂O₂ + engineered macrophage supernatant group, corresponding to 2.11-fold and 2.05-fold increases relative to the H₂O₂ group and the H₂O₂ + macrophage supernatant group, respectively, and reaching approximately 82% of the sham level. A similar pattern was observed for neurite outgrowth area. The sham group displayed the largest outgrowth area (17.75 ± 0.58 mm²), whereas the H₂O₂ group and the H₂O₂ + macrophage supernatant group showed markedly reduced areas of 5.85 ± 0.73 mm² and 5.93 ± 0.78 mm², respectively. Treatment with engineered macrophage-conditioned medium increased the outgrowth area to 11.80 ± 0.75 mm², representing approximately 2-fold increases relative to both injury groups and reaching 66% of the sham value (Figure 2D). These data indicate that engineered macrophages can markedly improve neurite/axonal outgrowth under oxidative stress conditions.

We next examined neuronal structural markers in PC-12 cells exposed to oxidative stress. Immunofluorescence staining showed that H₂O₂ markedly reduced NF, Tuj1, and MAP2 signals, whereas treatment with engineered macrophage-conditioned medium restored all three markers (Figure 2E, F). Quantitative analysis showed that the H₂O₂ group contained 40.67 ± 1.97% Tuj1-positive cells, 37.00 ± 2.58% NF-positive cells, and 21.83 ± 3.13% MAP2-positive cells. Similar values were observed in the H₂O₂ + macrophage supernatant group (40.83 ± 3.13%, 31.17 ± 2.56%, and 22.67 ± 2.94%, respectively), indicating limited protective activity of unmodified macrophage-conditioned medium under these conditions. In contrast, engineered macrophage-conditioned medium increased the proportions of Tuj1-, NF-, and MAP2-positive cells to 77.67 ± 3.88%, 81.17 ± 5.23%, and 79.17 ± 2.99%, respectively (Figure 2G-I). Compared with the H₂O₂ group, these values represented 1.91-fold, 2.19-fold, and 3.62-fold increases, respectively. Together, these results indicate that engineered macrophages more effectively preserve neuronal structural markers and neurite integrity under oxidative stress.

We then assessed whether engineered macrophage-conditioned medium affected PC-12 cells under basal conditions. Highly differentiated PC-12 cells were cultured in control medium, macrophage-conditioned medium, or engineered macrophage-conditioned medium without H₂O₂ challenge. Proliferation, apoptosis, and survival were evaluated by EdU labeling, TUNEL staining, and Calcein-AM/PI staining, respectively (Figure 2J-L). No significant differences were observed among the three groups under basal culture conditions. Specifically, proliferation rates were 91.33 ± 2.34%, 89.83 ± 2.64%, and 90.00 ± 3.16%, respectively (Figure 2M); apoptosis rates were 10.33 ± 1.86%, 9.83 ± 2.48%, and 9.33 ± 2.16%, respectively (Figure 2N); and survival rates were 99.33 ± 0.82%, 99.33 ± 0.75%, and 99.67 ± 0.52%, respectively (Figure 2O). These findings indicate that engineered macrophage-conditioned medium was well tolerated and did not exert detectable adverse effects on PC-12 cells under basal conditions.

Finally, we asked whether engineered macrophages could reduce neuronal injury under H₂O₂-induced oxidative stress. TUNEL staining showed that the apoptosis rate was 63.50 ± 3.83% in the H₂O₂ group and 63.83 ± 2.71% in the H₂O₂ + macrophage supernatant group, with no significant difference between them (Figure 2P, R). In contrast, the H₂O₂ + engineered macrophage supernatant group showed a markedly reduced apoptosis rate of 27.17 ± 2.99%, corresponding to approximately 42% of that observed in the two injury groups. Consistently, live/dead staining showed that cell survival remained below 70% in both the H₂O₂ group and the H₂O₂ + macrophage supernatant group, whereas survival increased to 83.3 ± 2.52% in the H₂O₂ + engineered macrophage supernatant group, representing 1.37-fold and 1.34-fold increases, respectively (Figure 2Q, S). These data indicate a clear protective effect of engineered macrophages against oxidative stress-induced neuronal injury.

Overall, engineered macrophages were well tolerated under basal conditions and exerted marked neuroprotective as well as neurite/axonal growth-promoting effects under oxidative stress *in vitro*. Although these effects did not fully restore all readouts to sham levels, they support the capacity of the engineered macrophage platform to improve neuronal survival and growth-related responses in an injury-relevant microenvironment.

These *in vitro* findings are consistent with the intended paracrine function of the engineered macrophages. ANXA1 may contribute to the suppression of inflammatory injury, GDNF provides neurotrophic support for neuronal survival and axonal extension, and CTGF may support matrix-associated repair processes. Although conditioned-medium experiments mainly reflect soluble-factor-mediated effects and cannot fully model the complexity of the injured spinal cord, they provide functional evidence that the engineered cells can generate a protective and growth-supportive secretory environment under oxidative stress.

### Engineered CCR2 positive macrophages alleviate after injury inflammation by shifting inflammatory cell phenotypes and cytokine profiles

**Figure 3:**
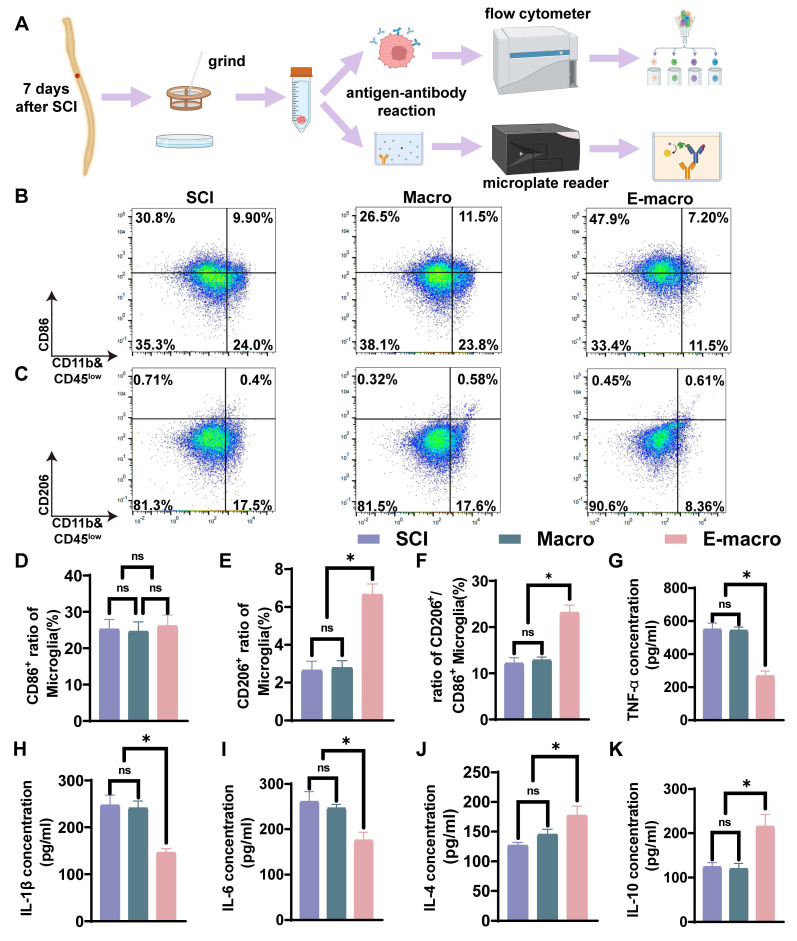
** Engineered macrophages reduce early inflammation by rebalancing inflammatory cell phenotypes and modulating cytokine expression *in vivo*.** (A) Schematic diagram of the workflow for flow cytometry and ELISA analysis. (B–C) Flow cytometric analysis of spinal cord tissue at the injury site, showing the proportions of CD11b⁺CD86⁺ cells (CD86-positive inflammatory phenotype-associated cells) and CD11b⁺CD206⁺ cells (CD206-positive reparative phenotype-associated cells). (D) Percentage of CD86⁺ cells. (E) Percentage of CD206⁺ cells. (F) Ratio of CD206⁺/CD86⁺ cells. (G–K) Quantification of inflammatory cytokines by ELISA, including TNF-α (G), IL-1β (H), IL-6 (I), IL-4 (J), and IL-10 (K). Macro: unmodified macrophage-treated group; E-macro: engineered CCR2⁺ macrophage-treated group. Data are presented as mean ± SD; n = 3 biologically independent animals per group. Statistical significance was determined by one-way ANOVA. *p < 0.05; ns, not significant.

SCI elicits a robust inflammatory response that contributes to neuronal and myelin loss. To investigate the immunomodulatory effects of engineered macrophages following SCI, we harvested spinal cord tissues from mice at day 7 post-injury and performed flow cytometric analysis together with cytokine quantification (Figure 3A). In the flow cytometric analysis, resident microglia and infiltrating macrophages were distinguished according to CD45 expression intensity, with CD45^low^&CD11b^+^ cells defined as microglia and CD45^high^CD11b^+^ cells defined as infiltrating macrophages (Figure S3A) 55. Quantitative analysis showed that the CD45^high^ infiltrating macrophage population accounted for only a minor fraction of total myeloid cells and did not differ significantly among groups (Figure S3B).

We next examined phenotype-associated markers within the CD45^high^ macrophage population. The proportion of CD86^+^ macrophages remained high and comparable among the SCI, macrophage-treated, and engineered macrophage-treated groups (Figure S3C). In contrast, the engineered macrophage-treated group showed a significantly higher proportion of CD206^+^ macrophages together with a marked increase in the CD206/CD86 ratio (Figure S3D, E). These data suggest that although infiltrating macrophages were relatively sparse at this time point, their phenotype also shifted toward a more anti-inflammatory state after engineered macrophage treatment.

Resident microglia constituted the predominant CD11b^+^CD45^low^ myeloid population in the injured spinal cord. The main figure therefore focuses on phenotypic changes within this compartment. Within the CD45^low^CD11b^+^ gate, the proportion of CD86^+^ pro-inflammatory microglia were comparable among the SCI group (25.25 ± 2.66%), the macrophage-treated group (24.63 ± 2.62%), and the engineered macrophage-treated group (26.15 ± 2.96%) (Figure 3B, D). By contrast, CD206^+^ anti-inflammatory microglia were present at low levels in both the SCI group (2.658 ± 0.47%) and the macrophage-treated group (2.792 ± 0.37%), with no significant difference between the two groups. However, in the engineered macrophage-treated group, this proportion increased significantly to 6.660 ± 0.5564%, representing 2.50-fold and 2.39-fold increases relative to the SCI and macrophage-treated groups, respectively (Figure 3C, E). Consistent with this shift, the CD206/CD86 ratio within the microglial population was markedly elevated in the engineered macrophage-treated group (23.17 ± 1.56%) compared with the SCI group (12.19 ± 1.19%) and the macrophage-treated group (12.87 ± 0.65%) (Figure 3F). Engineered macrophage treatment increased the proportion of CD206^+^ microglia and elevated the CD206/CD86 ratio within the lesion microenvironment.

To further characterize the local inflammatory milieu, we measured representative pro- and anti-inflammatory cytokines in spinal cord tissue. In the SCI and macrophage groups, pro-inflammatory cytokines IL-1β (247.0 ± 21.66 and 241.0 ± 15.00), IL-6 (261.0 ± 22.27 and 246.7 ± 8.15), and TNF-α (550.0 ± 38.51 and 543.3 ± 20.50) were highly elevated, while anti-inflammatory cytokines IL-4 (127.0 ± 5.00 and 145.3 ± 8.51) and IL-10 (124.3 ± 9.50 and 120.0 ± 12.00) remained low, with no significant differences between the two groups. By contrast, the engineered macrophage group exhibited significantly reduced levels of IL-1β (146.3 ± 8.62), IL-6 (175.7 ± 17.47), and TNF-α (267.3 ± 30.27), along with markedly increased IL-4 (177.3 ± 15.50) and IL-10 (215.3 ± 27.02) (p < 0.05; Figure 3G–K). These findings further demonstrate that engineered macrophages attenuate microglial activation, rebalance cytokine expression, and create a more favorable immunological niche for neural repair after SCI.

The lesion microenvironment shifted toward a less inflammatory state following engineered macrophage treatment. This change was accompanied by an increased proportion of CD206^+^ microglia, an elevated CD206/CD86 ratio, and corresponding alterations in inflammatory factor expression. Supplementary flow cytometric analysis further suggests that infiltrating CD45^high^ macrophages, although present at low abundance, also displayed a more anti-inflammatory phenotype after engineered macrophage treatment. These findings support an early immunomodulatory effect of engineered macrophages and suggest that they may help establish a more repair-supportive inflammatory microenvironment after SCI 56.

### Engineered macrophages reduce neuroinflammation and apoptosis with increased ANXA1/FPR2-associated signals after SCI

**Figure 4:**
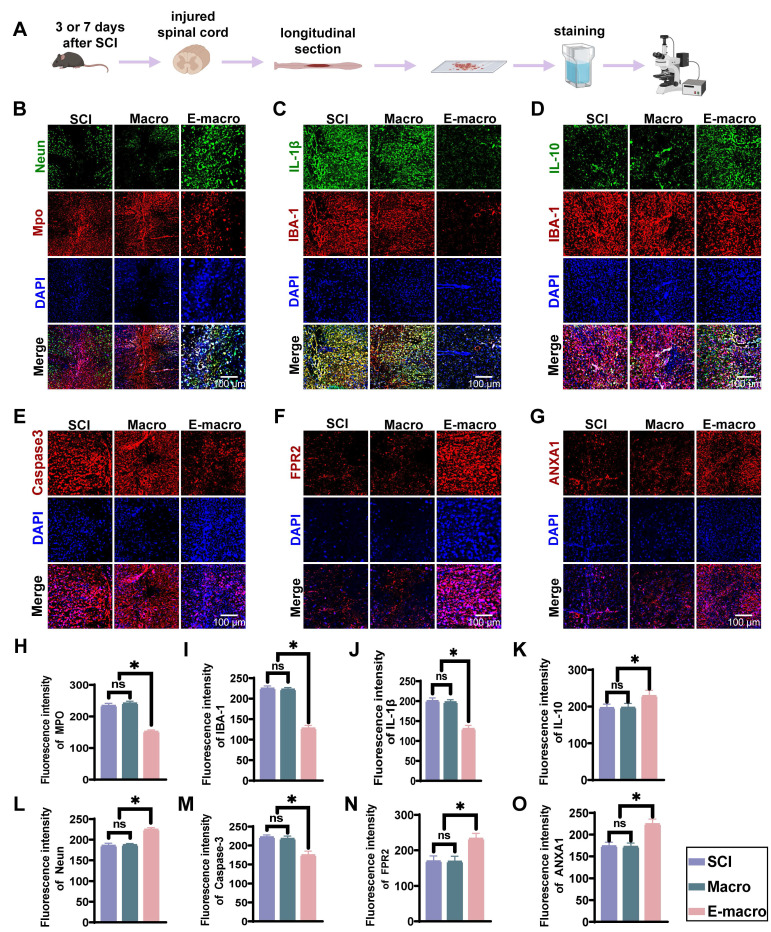
** Engineered macrophages are associated with reduced neuroinflammation, decreased apoptosis, and enhanced ANXA1/FPR2-related signaling after SCI.** (A) Experimental timeline: tissue collected on day 3 or day 7 after SCI for sectioning and immunofluorescence. (B) NeuN (green; neuronal nuclei marker), MPO (red; neutrophil marker), and DAPI. (Scale bar: 100 μm) (C) IL-1β (green), IBA-1 (red), and DAPI. (Scale bar: 100 μm) (D) Immunofluorescence of IL-10 (green), IBA-1 (red), and DAPI (blue). (Scale bar: 100 μm) (E) Caspase-3 (red; apoptosis marker) and DAPI. (Scale bar: 100 μm) (F) FPR2 (red; ANXA1 receptor) and DAPI. (Scale bar: 100 μm) (G) ANXA1 (red) and DAPI. (Scale bar: 100 μm). (H–O) Quantification of fluorescence intensity for MPO, IBA-1, IL-1β, IL-10, NeuN, Caspase-3, FPR2, and ANXA1 across groups. Data are presented as mean ± SD; n = 3 biologically independent animals per group. Statistical significance was determined by one-way ANOVA. *p < 0.05.

Given the ability of engineered macrophages to modulate inflammatory cell phenotypes and cytokine profiles, we next examined whether these cells were associated with changes in inflammatory cell activity, neuronal preservation, apoptosis, and ANXA1/FPR2-related signaling in injured spinal cord tissue (Figure 4A). In the acute stage of SCI (days 1–3), neutrophils rapidly infiltrate the lesion and are associated with disruption of the BSCB, and release proinflammatory mediators, triggering secondary damage that exacerbates neuronal loss and impairs tissue repair 57. To assess whether CCR2⁺ engineered macrophages could regulate neutrophil infiltration, the spinal cord was removed on the third day after the injury, and neutrophils were identified by MPO immunostaining (Figure 4B). Quantitative analysis demonstrated a significant reduction in MPO fluorescence intensity in the engineered macrophage group (151.8 ± 5.62) relative to the SCI group (233.8 ± 7.84) and the unmodified macrophage group (241.4 ± 6.98) (Figure 4H, *p* < 0.05). This reduction is consistent with decreased early neutrophil-associated inflammatory infiltration in the injured spinal cord after engineered macrophage treatment.

Microglia are important regulators of neuroinflammation and can shift toward different inflammatory states depending on the local microenvironment 58, 59. ANXA1 has been shown to modulate microglial polarization and spatial cytokine distribution, thereby exerting immunoregulatory and neuroprotective effects 60. In our flow cytometric analysis, CD45^low^ microglia were distinguished from CD45^high^ infiltrating macrophages, and the latter accounted for only a minor fraction of total myeloid cells at this stage. We therefore next examined lesion-associated IBA-1^+^ inflammatory cells and their cytokine-related features in spinal cord sections collected at day 7 after SCI. Immunofluorescence staining showed that IBA-1 fluorescence intensity in the engineered macrophage-treated group (127.5 ± 7.18) was significantly lower than that in the SCI group (224.9 ± 6.10) and the unmodified macrophage-treated group (222.4 ± 4.52) (Figure 4C–D, I; p < 0.01), indicating a reduction in IBA-1^+^ inflammatory cell signal after treatment. We then assessed the activation state of these IBA-1^+^ cells by quantifying cell body area, circularity, and process length (Figure S4A–C). Compared with the SCI and macrophage-treated groups, IBA-1^+^ cells in the engineered macrophage-treated group exhibited a smaller cell body area, lower circularity, and greater process length, consistent with a less activated and more ramified morphology. Because CD45^high^ infiltrating macrophages represented only a minor fraction of total myeloid cells at this time point, these morphological changes are likely to mainly reflect modulation of the microglia-dominant inflammatory compartment. However, since IBA-1 staining alone does not fully distinguish resident microglia from infiltrating macrophages, this interpretation should be made with appropriate caution.

Cytokine analysis further revealed that the engineered macrophage group had the lowest expression of IL-1β (120.7 ± 8.76), representing only 60% of that in the SCI (200.4 ± 7.59) and macrophage (197.2 ± 6.12) groups (*p* < 0.01). Conversely, IL-10 expression was highest in the engineered macrophage group (228.8 ± 15.28), markedly exceeding levels in the SCI (196.4 ± 10.18) and macrophage (197.2 ± 11.13) groups (*p* < 0.01; Figure 4J–K), suggesting a shift in the inflammatory milieu toward an anti-inflammatory state.

Considering that inflammation contributes to secondary neuronal apoptosis, we further assessed cell death using caspase-3 staining. At day 7 post-injury, the caspase-3 fluorescence intensity was lower in the engineered macrophage group (174.5 ± 10.66) than in the SCI (222.1 ± 5.92) and macrophage (217.7 ± 7.37) groups (*p* < 0.01; Figure 4E, M), corresponding to an approximate 25% reduction in apoptosis. Additionally, NeuN staining revealed higher neuronal preservation in the engineered macrophage group (224.6 ± 4.62), compared to the SCI (185.6 ± 5.78) and macrophage (187.2 ± 3.54) groups (*p* < 0.01; Figure 4B, L).

To better understand the underlying mechanism, we assessed the expression of ANXA1 and its downstream receptor FPR2. The engineered macrophage group showed significantly elevated levels of ANXA1 (224.4 ± 11.41) and FPR2 (232.9 ± 14.95) relative to the SCI (173.6 ± 8.92; 169.0 ± 15.06) and macrophage (172.1 ± 8.65; 168.2 ± 14.88) groups (*p* < 0.01; Figure 4F–G, N–O), corresponding to 1.29–1.38-fold increases.

In summary, engineered macrophage treatment was associated with a reduction in early inflammatory responses, reduced lesion-associated IBA-1⁺ inflammatory cell signal, a more favorable local cytokine profile, decreased apoptosis, and improved neuronal preservation after SCI. In parallel, increased ANXA1 and FPR2 expression in the lesion is consistent with participation of ANXA1/FPR2-related signaling in this process. Together with the flow cytometric evidence that CD45^high^ infiltrating macrophages account for only a minor proportion of myeloid cells at this stage, these findings support the view that engineered macrophage treatment exerts mainly microglia-associated immunomodulatory and neuroprotective effects in the injured spinal cord.

These results further support the anti-inflammatory and neuroprotective role of engineered macrophages *in vivo*. Because neutrophil infiltration, microglia/macrophage activation, and inflammatory cytokine production are closely linked to secondary neuronal injury after SCI 5, 6, 57, 58, 61, the concomitant reduction in MPO, IBA-1, IL-1β, and caspase-3 signals, together with increased IL-10 and ANXA1/FPR2-related staining, suggests that engineered macrophages may attenuate inflammatory amplification while preserving neural cells within the lesion microenvironment.

### Transcriptomic remodeling after engineered macrophage treatment

**Figure 5:**
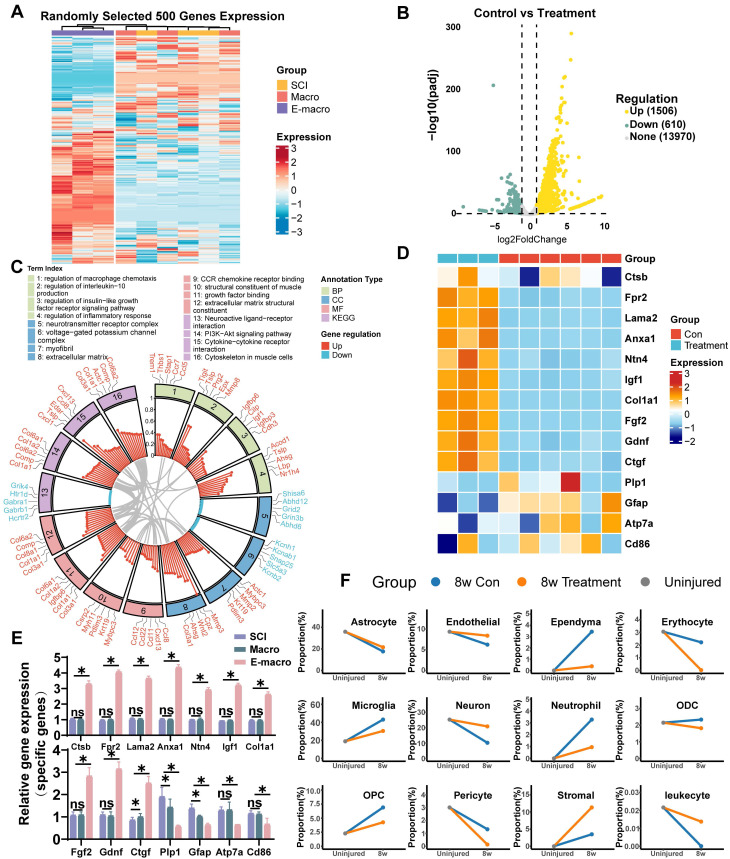
** Transcriptomic reprogramming and cell composition changes induced by engineered macrophages 8 weeks after SCI.** (A) Heatmap showing hierarchical clustering of 500 randomly selected gene expression profiles. (B) Volcano plot showing differentially expressed genes between the engineered macrophage group and the control group, using the thresholds |log₂FC| > 1.5 and p < 0.05. (C) Gene Ontology (GO) enrichment analysis of the differentially expressed genes, including biological process (BP), molecular function (MF), and cellular component (CC) categories. (D) Heatmap of 14 representative DEGs related to neural repair, inflammation regulation, and extracellular matrix remodeling. (E) Differential expression profiles of 14 key genes associated with neuroregeneration, modulation of inflammation, and extracellular matrix remodeling. (F) Deconvolution analysis based on mRNA sequencing data predicting the relative proportions of major cell types in spinal cord tissue across groups. RNA-seq was performed with n = 3 biologically independent samples per group. Differentially expressed genes were identified using |log₂FC| > 1.5 and p < 0.05.

To examine transcriptional remodeling in the chronic lesion environment, we performed RNA sequencing on tissue collected from the lesion core at 8 weeks after SCI. Genes meeting the criteria of |log₂FC| > 1.5 and p < 0.05 were defined as differentially expressed genes (DEGs). Hierarchical clustering showed that the SCI group and the unmodified macrophage-treated group shared broadly similar transcriptional profiles and were therefore combined as a single control group for comparison with the engineered macrophage-treated group (Figure 5A). In total, 2,116 DEGs were identified, including 1,506 upregulated and 610 downregulated genes (Figure 5B). Enrichment analysis highlighted biological processes and pathways related to inflammatory regulation, macrophage chemotaxis, extracellular matrix (ECM) organization, receptor signaling, and neural repair (Figure 5C). We next performed gene set enrichment analysis (GSEA) to further define pathway-level changes relevant to the therapeutic design of this study. Compared with controls, the engineered macrophage-treated group showed significant enrichment of pathways related to regulation of inflammatory response, regulation of macrophage chemotaxis, extracellular matrix structural constituent, PI3K-Akt signaling, and cytokine-cytokine receptor interaction (Figure S5A-E). Overall, these findings point to coordinated changes in inflammation, chemotaxis-related signaling, and matrix remodeling within the repaired spinal cord microenvironment. qPCR further supported the RNA-seq results. Several genes associated with neuroprotection and repair, including *Fgf2, Igf1, Ntn4, and Gdnf,* were significantly upregulated in the engineered macrophage-treated group. This expression pattern is consistent with a more supportive local environment for neuronal survival and axon-related growth. Increased expression of *Anxa1* and its receptor *Fpr2*, together with reduced *Cd86*, further suggested a less pro-inflammatory lesion milieu. In parallel, upregulation of *Col1a1*, *Ctgf*, and *Lama2* was consistent with enhanced ECM synthesis and remodeling, which may contribute structural support for tissue repair. By contrast, lower expression of *Gfap* and *Plp1* suggested attenuation of reactive gliosis and altered myelin-associated transcriptional status. Reduced *Atp7a* expression may likewise reflect lower copper-related stress responses, including oxidative stress, inflammation, and mitochondrial dysfunction (Figure 5D-E).

To estimate changes in tissue cellular composition, we applied BayesPrism-based deconvolution using published single-cell RNA-seq datasets as references 53, 54. This analysis suggested relatively higher proportions of neurons, endothelial cells, and stromal cells in the engineered macrophage-treated group, together with lower proportions of inflammatory populations such as microglia and neutrophils (Figure 5F). This pattern is consistent with a lesion environment characterized by reduced inflammatory burden and stronger structural support.

Overall, the transcriptomic and deconvolution data suggest that engineered macrophage treatment was associated with broad remodeling of the chronic SCI lesion environment. The observed changes were consistent with reduced inflammatory activity, altered chemotaxis-related signaling, enhanced ECM-related programs, and increased expression of genes linked to neural repair and tissue support. Together, these findings support the view that engineered macrophages can reshape the injured spinal cord microenvironment at both molecular and cellular levels.

These transcriptomic findings suggest that the therapeutic effect of engineered macrophages is not restricted to a single molecular pathway. Instead, the treatment was associated with coordinated changes in immune regulation, macrophage chemotaxis, cytokine signaling, neural repair, myelination, and extracellular matrix organization. This pattern is consistent with the multifactorial nature of SCI, in which inflammation, insufficient trophic support, inhibitory matrix remodeling, demyelination, and impaired axonal growth act together to limit recovery 5-7. Thus, the RNA-seq data provide molecular support for the histological and functional improvements observed in later experiments.

### Engineered macrophages accumulate at the lesion site and enhance GDNF signaling after SCI

Biodistribution analysis is important for assessing the *in vivo* lesion-associated accumulation of engineered cells. Precisely directing engineered macrophages to the site of SCI is expected to enhance therapeutic efficacy. To assess whether CCR2 enrichment was associated with enhanced accumulation of engineered macrophages at the lesion site, we performed *in vivo* fluorescence tracking experiments. DiR-labeled macrophages or CCR2-enriched engineered macrophages were intravenously injected into SCI model mice, and their biodistribution was monitored over time.

**Figure 6:**
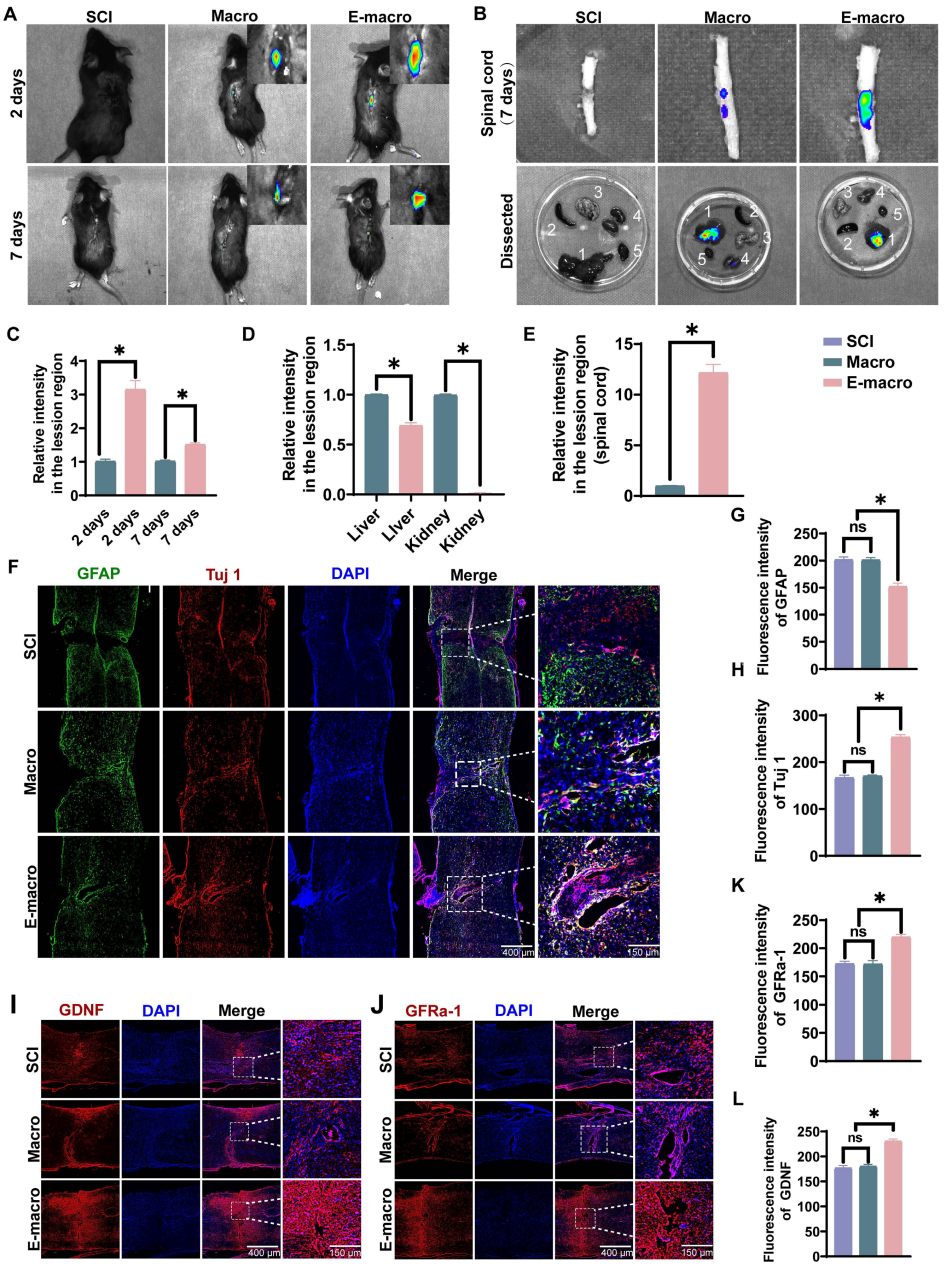
** Engineered macrophages show enhanced lesion-associated accumulation and increase GDNF/GFRα-1-associated signals after SCI.** (A) Representative *in vivo* fluorescence images showing biodistribution of DiR-labeled macrophages and engineered macrophages at 2 and 7 days after systemic administration in SCI mice. (B) Representative *ex vivo* fluorescence images of isolated spinal cords and major dissected organs at 7 days after administration. Organs were labeled as follows: 1. Liver; 2. Spleen; 3. Lungs; 4. Kidneys; 5. Heart. (C) Quantification of fluorescence intensity in the lesion region at 2 and 7 days after injection. (D) Quantification of fluorescence intensity in the liver and kidney. (E) Quantification of fluorescence intensity in isolated spinal cord tissue at 7 days after injection. (F) Representative immunofluorescence images of GFAP (green), Tuj1 (red), and DAPI (blue) in injured spinal cord sections. Enlarged views of the boxed regions are shown on the right. (Scale bars: 400 μm for overview images and 150 μm for magnified images.) (G–H) Quantification of fluorescence intensities of GFAP and Tuj1, respectively. (I) Representative immunofluorescence staining of GDNF (red) and DAPI (blue) in injured spinal cord sections. Enlarged views of the boxed regions are shown on the right. (Scale bars: 400 μm for overview images and 150 μm for magnified images.) (J) Representative immunofluorescence staining of GFRα-1 (red) and DAPI (blue) in injured spinal cord sections. Enlarged views of the boxed regions are shown on the right. (Scale bars: 400 μm for overview images and 150 μm for magnified images.) (K–L) Quantification of fluorescence intensities of GDNF and GFRα-1, respectively. Data are presented as mean ± SD; n = 3 animals per group for biodistribution analysis and n = 6 animals per group for immunofluorescence analysis. Statistical significance was determined by one-way ANOVA. *p < 0.05.

Fluorescence imaging at day 2 post-injury showed that the engineered macrophage group had a markedly stronger signal in the spinal lesion region than the unmodified macrophage group (3.156 ± 0.26 vs. 1.01 ± 0.07; p < 0.01), corresponding to a 3.11-fold increase (Figure 6A, C). By day 7, fluorescence intensity had declined in both groups, but the engineered macrophage group still retained a stronger signal than the unmodified macrophage group (1.514 ± 0.05 vs. 1.012 ± 0.04), corresponding to a 1.49-fold difference (Figure 6A, C). *Ex vivo* imaging of isolated spinal cords further supported the same trend, showing that fluorescence intensity at the lesion site in the engineered macrophage group was approximately 15-fold higher than that in the unmodified macrophage group (Figure 6B, E). Analysis of major organs further showed that unmodified macrophages predominantly accumulated in the liver and kidneys, whereas engineered macrophages exhibited significantly reduced signals in these organs—liver fluorescence was only 70% of that in the control group (Figure 6B, D). These results are consistent with enhanced lesion-associated accumulation of engineered macrophages after systemic administration, although they do not, on their own, establish a strictly receptor-dependent targeting mechanism.

We next examined glial scar-associated remodeling and neuronal structural changes by GFAP and Tuj1 immunofluorescence staining (Figure 6F). GFAP fluorescence intensity was comparable between the SCI group (201.8 ± 4.99) and the macrophage-treated group (201.3 ± 4.21), but was significantly reduced in the engineered macrophage-treated group (152.2 ± 5.94), corresponding to approximately 75% of the level observed in the other two groups (Figure 6G). In contrast, Tuj1 fluorescence intensity was markedly higher in the engineered macrophage-treated group (253.3 ± 5.38) than in the SCI group (166.9 ± 5.14) and the macrophage-treated group (170.9 ± 1.79), representing 1.52-fold and 1.48-fold increases, respectively (Figure 6H). This pattern is consistent with reduced scar-associated change together with improved preservation of neuronal structural signal after engineered macrophage treatment.

We then asked whether these local tissue changes were accompanied by altered neurotrophic signaling. At 8 weeks after SCI, immunofluorescence staining showed that both GDNF and GFRα-1 were significantly increased in the engineered macrophage-treated group. Fluorescence intensities of GDNF and GFRα-1 reached 230.8 ± 4.17 and 219.8 ± 4.75, respectively, compared with 177.0 ± 4.87 and 172.7 ± 4.16 in the SCI group, and 180.2 ± 3.86 and 172.0 ± 6.09 in the macrophage-treated group (Figure 6I-L). These findings are consistent with enhanced GDNF/GFRα-1-related signaling within the lesion after engineered macrophage treatment.

Overall, engineered macrophages showed greater lesion-associated accumulation after systemic administration and were associated with increased neurotrophic signaling, reduced GFAP-positive scar-associated change, and improved neuronal structural preservation in the injured spinal cord.

These findings suggest that the therapeutic activity of engineered macrophages may depend on both lesion-associated accumulation and local cargo expression. Compared with passive delivery, CCR2-enriched macrophages can exploit the CCL2-rich inflammatory environment after SCI, thereby improving delivery to the injured spinal cord 26-28. The increase in GDNF/GFRα-1-related staining further supports the neurotrophic component of the platform, while the reduction in GFAP-associated signal indicates that engineered macrophage treatment may help attenuate scar-associated barriers to axonal repair 43-46.

### Engineered macrophages are associated with extracellular matrix remodeling and enhanced axon associated repair in SCI

The extracellular matrix (ECM) is important for neural repair because it provides structural support for axonal growth. Connective tissue growth factor (CTGF), a member of the CCN family, functions as a downstream mediator of TGF-β signaling and mediates its profibrotic effects, thereby promoting ECM synthesis and remodeling 62-64. To determine whether engineered macrophage treatment was associated with a more growth-supportive ECM environment in SCI, we performed immunofluorescence staining at 8 weeks after SCI.

**Figure 7:**
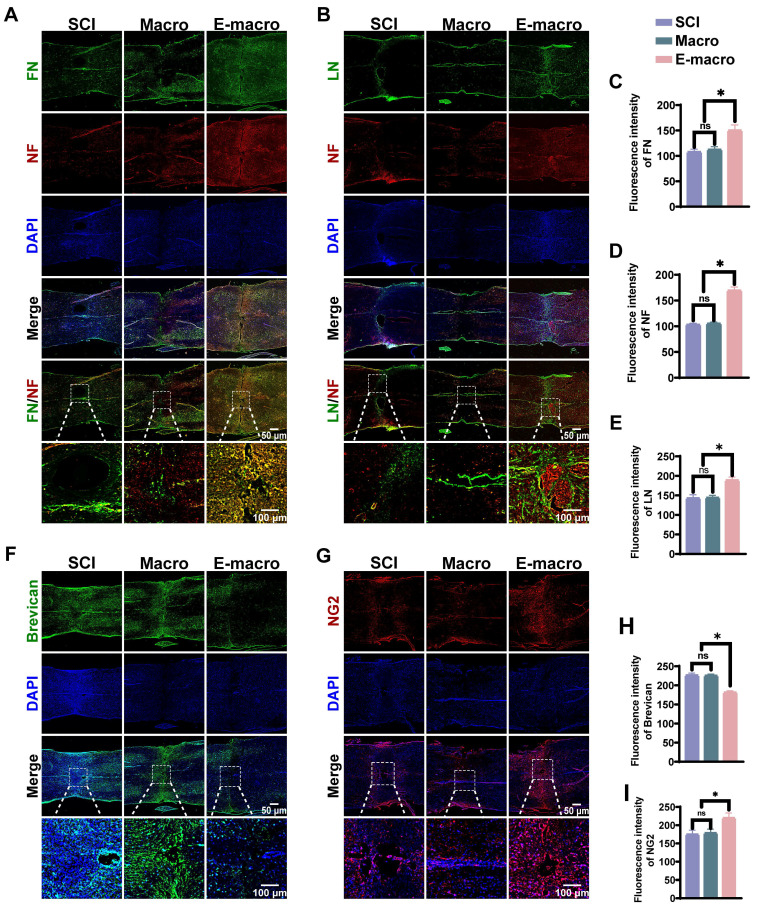
** Engineered macrophages are associated with extracellular matrix remodeling and enhanced axon-associated repair in SCI.** (A) Representative immunofluorescence images of the lesion area showing expression of fibronectin (FN, green), neurofilament (NF, red), and nuclei (DAPI, blue); lower panels display magnified views of FN/NF colocalization. (Scale bars: 50 μm for overview images and 100 μm for magnified images.) (B) Representative immunofluorescence images showing laminin (LN, green), NF (red), and DAPI (blue) in the lesion area; lower panels show magnified LN/NF colocalization. (Scale bars: 50 μm for overview images and 100 μm for magnified images.) (C–E) Quantification of average fluorescence intensity for FN, NF, and LN across groups. (F) Immunofluorescence staining of brevican (green, inhibitory CSPG) and DAPI (blue); lower panels show magnified lesion areas. (Scale bars: 50 μm for overview images and 100 μm for magnified images.) (G) Immunostaining of NG2 (red, CSPG-associated marker) and DAPI (blue); lower panels show magnified views. (Scale bars: 50 μm for overview images and 100 μm for magnified images.) (H–I) Quantification of average fluorescence intensity of brevican and NG2 in each group. Data are presented as mean ± SD; n = 6 biologically independent animals per group. Statistical significance was determined by one-way ANOVA. *p < 0.05.

In the engineered macrophage group, neurofilament (NF) signal showed greater overlap with the ECM proteins fibronectin (FN) and laminin (LN) within the lesion area, compared with the SCI and unmodified macrophage groups (Figure 7A, B). In the engineered macrophage group, the average fluorescence intensity reached 150.5 ± 10.48 for FN and 189.2 ± 7.72 for LN, respectively—markedly greater than those in the macrophage group (112.6 ± 5.53 and 144.5 ± 5.69) and the SCI group (107.9 ± 5.39 and 143.2 ± 8.66) (*p* < 0.01). No obvious difference was observed between the SCI and macrophage groups (*p* > 0.05) (Figure 7C, E). Further analysis revealed that FN and LN levels in the engineered macrophage group were approximately 1.39-fold and 1.32-fold higher, respectively, compared to the SCI group, suggesting enhanced ECM deposition and structural support for axonal regrowth. These findings suggest that engineered macrophage treatment promoted a lesion matrix environment more favorable for repair.

Astrocyte-derived chondroitin sulfate proteoglycans (CSPGs) are important extracellular matrix components known to influence axonal plasticity after SCI 6, 65. We therefore examined NG2 and brevican expression in the lesion area to determine whether engineered macrophage treatment was associated with altered CSPG composition (Figure 7F, G). Brevican expression was significantly lower in the engineered macrophage-treated group (181.7 ± 4.07) than in the SCI group (226.5 ± 5.45) and the macrophage-treated group (225.5 ± 3.95), reaching approximately 80% of the levels observed in the two control groups. In contrast, NG2 expression was significantly higher in the engineered macrophage-treated group (220.1 ± 13.25), corresponding to a 1.23-fold increase relative to the SCI group (175.0 ± 11.42) and the macrophage-treated group (178.5 ± 10.51) (Figure 7H, I). Together, these changes suggest that engineered macrophage treatment altered the lesion-associated CSPG profile and may be associated with matrix remodeling and repair-related cellular responses.

We next asked whether these ECM-related changes were accompanied by improved axon-associated structural signals. Neurofilament (NF) fluorescence intensity was similar in the SCI group and the macrophage-treated group (103.2 ± 2.60 and 105.4 ± 2.70, respectively), with no significant difference between them. In contrast, NF intensity was markedly higher in the engineered macrophage-treated group (169.8 ± 6.38), representing 1.65-fold and 1.61-fold increases relative to the SCI and macrophage-treated groups, respectively (Figure 7A, B, D). This pattern is consistent with improved axon-associated structural preservation or repair after engineered macrophage treatment.

Overall, engineered macrophages were associated with increased expression of growth-supportive ECM components, reduced inhibitory matrix-associated signals, and stronger NF-positive axon-related signal within the lesion. Rather than pointing to a single downstream mechanism, these findings support a broader role for engineered macrophages in establishing a more permissive extracellular environment for axon-associated repair after SCI.

### Functional assessment of axon associated structural repair and remyelination after engineered macrophage treatment

**Figure 8:**
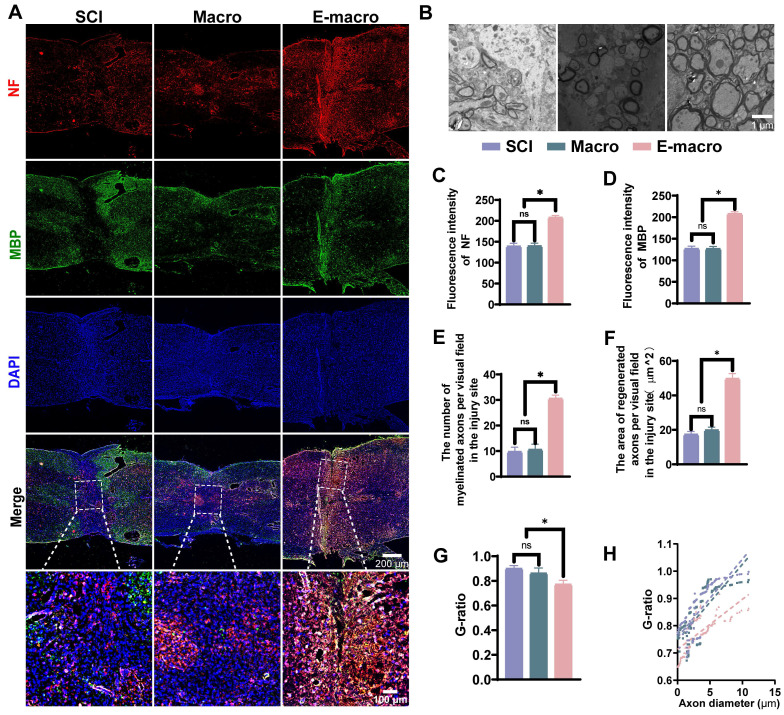
Engineered macrophages are associated with enhanced axon-associated structural repair and remyelination at the injury site. (A) Representative immunofluorescence images of spinal cord sections stained for NF (red), MBP (green), and DAPI (blue). Enlarged images of the boxed regions are shown below. (Scale bars: 50 μm for overview images and 100 μm for magnified images.) (B) Representative TEM images obtained at 8 weeks after injury, showing regenerated axons and surrounding myelin sheaths. (Scale bar: 1 μm). (C–D) Quantification of NF and MBP fluorescence intensities in the lesion area, respectively. (E) Quantification of the average number of regenerated axons per field at 8 weeks post-injury. (F) Quantification of the mean cross-sectional area of regenerated axons. (G) Comparison of the g-ratio among groups. (H) Scatter plot showing the relationship between g-ratio and axon diameter in each group at 8 weeks after injury. Data are presented as mean ± SD; n = 6 biologically independent animals per group. Statistical significance was determined by one-way ANOVA. *p < 0.05.

It has been reported that the restoration of motor function after SCI largely depends on the extent of axonal regeneration 65-67. To evaluate the pro-regenerative effects of engineered macrophages, we used neurofilament (NF) as a marker to assess axonal growth within the lesion area. Notably, the engineered macrophage group exhibited dense clusters of NF-positive axons at the injury site, with an average fluorescence intensity of 58.92 ± 8.27—representing a 6.88-fold and 6.49-fold increase compared to the SCI group (7.74 ± 1.25) and the unmodified macrophage group (8.26 ± 1.33) (p < 0.01; Figure 8A, C). Because effective remyelination is important for restoring rapid signal conduction after SCI, we next evaluated myelin-related repair by staining for myelin basic protein (MBP) (Figure 8A). Strong MBP-positive signals were observed in both the lesion core and peri-lesional regions in the engineered macrophage group, whereas the SCI and macrophage groups showed only weak MBP staining. Quantitative analysis showed that MBP fluorescence intensity in the engineered macrophage group reached 106.09 ± 18.22, which was 12.14-fold and 11.46-fold higher than that in the SCI group (8.74 ± 1.25) and macrophage group (9.26 ± 1.33), respectively (Figure 8D). These findings indicate a marked improvement in remyelination-related repair after engineered macrophage treatment.

We then used transmission electron microscopy (TEM) to evaluate ultrastructural changes at 8 weeks after SCI (Figure 8B). The number of regenerated axons per visual field was significantly higher in the engineered macrophage-treated group (30.50 ± 1.38) than in the SCI group (9.67 ± 1.86) and the macrophage-treated group (10.50 ± 2.17), representing 3.16-fold and 2.90-fold increases, respectively (Figure 8E). The mean cross-sectional area of regenerated axons also increased to 49.70 ± 2.96 μm² in the engineered macrophage-treated group, compared with 17.27 ± 1.80 μm² in the SCI group and 19.78 ± 1.78 μm² in the macrophage-treated group (Figure 8F). This pattern is consistent with reduced axonal atrophy. TEM images further showed that axons in the engineered macrophage-treated group were more frequently ensheathed by thick and compact myelin. In line with this observation, the mean g-ratio decreased to 0.77 ± 0.03, compared with 0.90 ± 0.02 in the SCI group and 0.86 ± 0.04 in the macrophage-treated group (Figure 8G, H). By contrast, many axons in the SCI group remained poorly myelinated or unmyelinated, indicating limited spontaneous remyelination. Overall, these findings suggest that engineered macrophage treatment improved both the extent and the quality of myelin-related repair.

Collectively, engineered macrophages were associated with stronger axon-related structural signals, increased numbers and size of regenerated axons, and improved remyelination-related ultrastructural features in the injured spinal cord. These results support a beneficial effect of engineered macrophage treatment on axon-associated structural repair and remyelination after SCI.

### Engineered macrophages enhance synaptogenesis and promote sensorimotor circuit reconstruction

**Figure 9:**
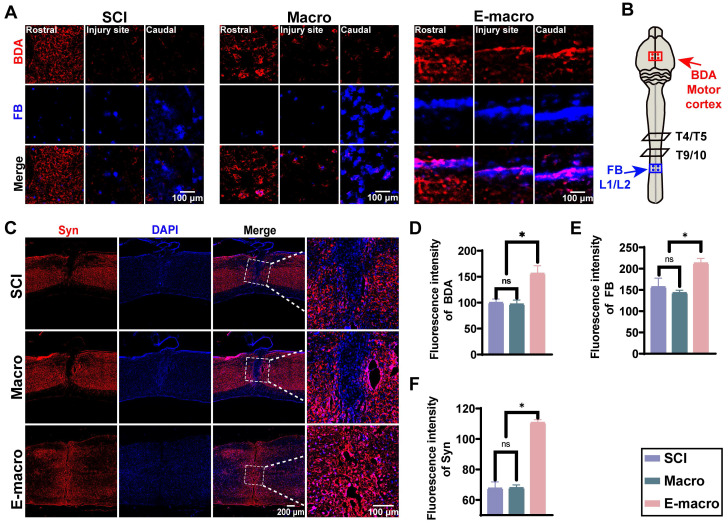
** Engineered macrophages facilitate synapse formation and functional neural circuit reconstruction after SCI.** (A) Representative immunofluorescence staining of the lesion site showing BDA-labeled corticospinal tract (CST) axons (red) and Fast Blue (FB)-positive sensory neurons (blue), reflecting axonal projection and sensory circuit connectivity. (Scale bar: 100 μm). (B) Schematic diagram showing the injection locations of BDA and FB for anterograde and retrograde tracing. (C) Representative images of Synaptophysin (Syn, red) staining in the lesion area, with DAPI (blue) used for nuclear counterstaining, indicating synapse formation in the injured spinal cord. (Scale bars: 50 μm for overview images and 100 μm for magnified images.) (D–F) Quantitative analysis of the average fluorescence intensity of BDA, FB and Syn in the injury area of each group. Data are presented as mean ± SD; n = 6 biologically independent animals per group. Statistical significance was determined by one-way ANOVA. *p < 0.05.

At 8 weeks after SCI, we examined synapse-related changes by immunofluorescence staining for synaptophysin (Syn), a presynaptic marker. The engineered macrophage-treated group showed a denser distribution of Syn-positive puncta within the lesion core than the SCI group and the unmodified macrophage-treated group (Figure 9C). Quantitatively, Syn fluorescence intensity reached 110.8 ± 1.94 in the engineered macrophage-treated group, compared with 67.62 ± 4.21 in the SCI group and 67.94 ± 1.92 in the macrophage-treated group, representing 1.64-fold and 1.63-fold increases, respectively (Figure 9F). This pattern suggests improved synapse-associated structural preservation after engineered macrophage treatment. Since SCI disrupts both axonal continuity and synaptic connectivity, the increase in Syn-positive signal may help support later recovery of neural signal transmission. Thus, re-establishing the structural integrity of both sensory and motor pathways is critical for functional recovery. To further assess the contribution of engineered macrophages to circuit rewiring, we performed both anterograde and retrograde tract tracing. BDA was injected into the motor cortex for anterograde tracing of corticospinal tract (CST) axons, whereas Fast Blue (FB) was injected bilaterally 5–6 mm caudal to the lesion to retrogradely label ascending sensory axons. At the proximal site, BDA labeling of CST axons was comparable among all groups. However, in the lesion and distal segments, BDA-positive fibers were rarely observed in the SCI and unmodified macrophage groups, indicating limited axonal regeneration across the lesion. In contrast, robust BDA labeling was evaluated in the distal spinal cord of mice treated with engineered macrophages. Quantitative analysis showed that BDA fluorescence intensity at the lesion site reached 156.3 ± 15.05 in the engineered macrophage group, which was 1.56 times that of the SCI group (100.1 ± 6.86) and 1.61 times that of the macrophage group (97.08 ± 7.82) (Figure 9A, D). A similar trend was observed in FB retrograde tracing. While no significant differences were noted in the distal spinal cord, FB-labeled neurons were nearly absent in the lesion and proximal regions of the SCI and macrophage groups. In contrast, the engineered macrophage group showed a substantial increase in FB-positive neurons rostral to the lesion, suggesting successful reconnection of ascending sensory axons. Specifically, the FB signal intensity at the lesion site in this group was 213.0 ± 11.04, which was 1.35-fold and 1.49-fold higher than that in the SCI (157.2 ± 20.35) and macrophage groups (143.2 ± 6.12), respectively (Figure 9A, E).

Overall, engineered macrophage treatment was associated with stronger synapse-related signal and improved axon tracing across the lesion, changes that may help support neural repair after SCI. This pattern may be linked to greater lesion-associated accumulation of engineered macrophages, along with local changes in inflammatory regulation, neurotrophic support, and extracellular matrix remodeling. Although the current data do not define a complete circuit-reconstruction mechanism, they support the potential of engineered macrophages as a cell-based approach for structural repair and partial functional improvement after SCI.

These structural changes are consistent with the tri-modal design of the engineered macrophage platform. CTGF is closely related to extracellular matrix production and remodeling, while ECM components such as fibronectin and laminin can provide a more permissive substrate for axonal extension and tissue repair 68. In parallel, increased NF, MBP, BDA, FB, and synaptophysin signals suggest that engineered macrophage treatment was associated with axon-related repair, remyelination, and partial reconstruction of neural connectivity 69, 70. These findings do not prove complete circuit restoration, but they support a coordinated tissue-repair response involving matrix remodeling, axonal preservation or regrowth, myelin-associated recovery, and synaptic remodeling.

### Engineered macrophages alleviate muscle atrophy and enhance electromyographic activity following SCI

**Figure 10:**
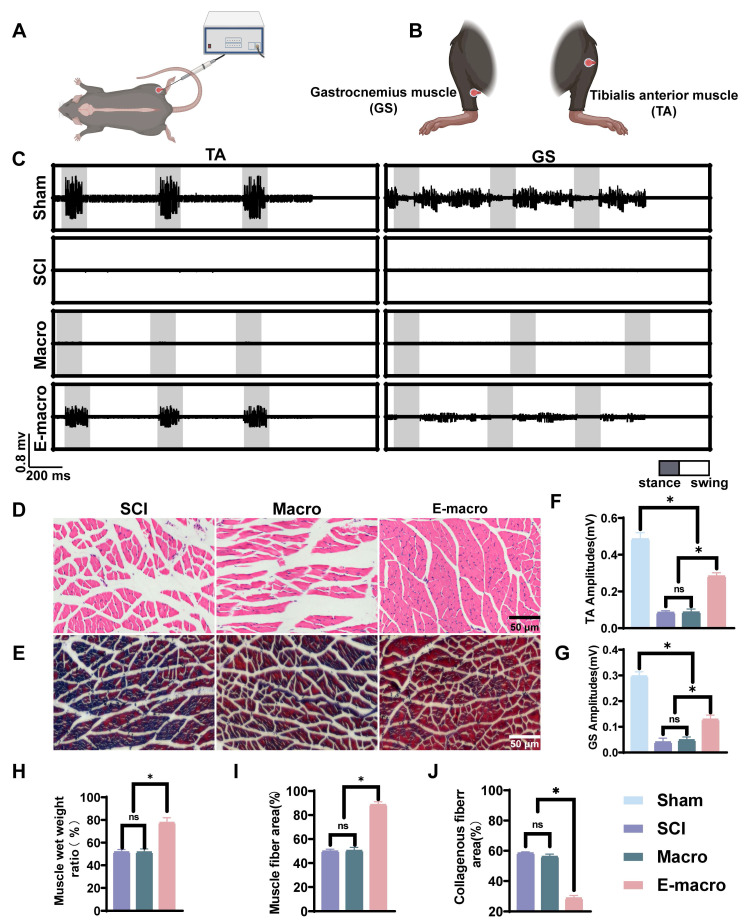
** Intravenous administration of engineered macrophages alleviates muscle atrophy and improves electromyographic activity.** (A–B) Schematic illustration of muscle electrophysiological recordings 8 weeks after spinal cord injury (SCI). (C) Representative EMG traces of gastrocnemius–soleus (GS) and tibialis anterior (TA) muscles in mice from different treatment groups. (D–E) Representative images of H&E staining and Masson’s trichrome staining of gastrocnemius muscles at 8 weeks post-SCI (scale bar: 50 μm). (F–G) Quantification of mean EMG amplitudes of TA and GS muscles (in mV). (H) Wet weight of gastrocnemius muscle. (I) Percentage of muscle fiber area. (J) Percentage of collagen fiber area. Data are presented as mean ± SD; n = 4 biologically independent animals per group for EMG analysis and n = 6 biologically independent animals per group for muscle wet weight and histological analysis. Statistical significance was determined by one-way ANOVA. *p < 0.05; ns, not significant.

We next assessed hindlimb muscle activity by electromyography (EMG) at 8 weeks after SCI, as muscle output provides a useful functional readout of neuromuscular recovery. In both the SCI group and the unmodified macrophage-treated group, the tibialis anterior (TA) and gastrocnemius-soleus (GS) muscles showed weak and irregular activation, and coordinated alternating discharge during locomotion was rarely observed. By contrast, mice treated with engineered macrophages displayed clearer alternating activation of the TA and GS muscles throughout the gait cycle (Figure 10C). Their EMG waveforms were also better organized and of higher amplitude, approaching the pattern observed in sham animals. Together, these features are consistent with improved neuromuscular control.

Quantitative analysis further supported these observations. In the engineered macrophage-treated group, the mean TA amplitude reached 0.71 ± 0.12 mV, compared with 0.12 ± 0.03 mV in the SCI group and 0.13 ± 0.02 mV in the unmodified macrophage-treated group, corresponding to 5.92-fold and 5.47-fold increases, respectively. For the GS muscle, the mean amplitude was 0.28 ± 0.05 mV, compared with 0.09 ± 0.01 mV in the SCI group and 0.10 ± 0.01 mV in the unmodified macrophage-treated group, representing 3.1-fold and 2.8-fold increases, respectively. Although these values did not fully return to sham levels (TA: 1.26 ± 0.27 mV; GS: 0.61 ± 0.08 mV), they reached 56% and 46% of normal values, respectively (p < 0.01), indicating substantial recovery of motor output and muscle coordination (Figure 10F, G).

We then examined whether this functional improvement was accompanied by better preservation of muscle structure. Histological analysis of the gastrocnemius muscle showed marked atrophy in both the SCI group and the unmodified macrophage-treated group, characterized by disorganized myofiber arrangement, widened interstitial spaces, inflammatory infiltration, and excessive collagen deposition on H&E and Masson’s trichrome staining. In contrast, the engineered macrophage-treated group showed markedly improved muscle morphology, including more orderly myofiber organization, reduced interstitial expansion, less inflammatory infiltration, and lower collagen deposition. These findings suggest that engineered macrophage treatment not only improves muscle activity, but also helps preserve skeletal muscle structure after SCI. Muscle area quantification showed that the engineered macrophage group reached 88.61 ± 1.13%, compared to 50.40 ± 2.63% and 49.88 ± 1.66% in the macrophage and SCI groups, respectively (p < 0.01). The collagen fiber area ratio in the engineered macrophage group was 28.67 ± 1.74%, markedly lower than that of the macrophage group (56.13 ± 1.66%) and SCI group (58.46 ± 0.88%) (p < 0.01, Figure 10H–J).

Collectively, these results demonstrate that engineered macrophages significantly enhance neuromuscular function following SCI by improving muscle activation and coordination, while simultaneously mitigating muscle atrophy and collagen overaccumulation. These effects collectively contribute to early remodeling of motor circuits and functional recovery.

### Engineered macrophages improve conduction-related electrophysiological signals and promote partial motor functional recovery after SCI

**Figure 11:**
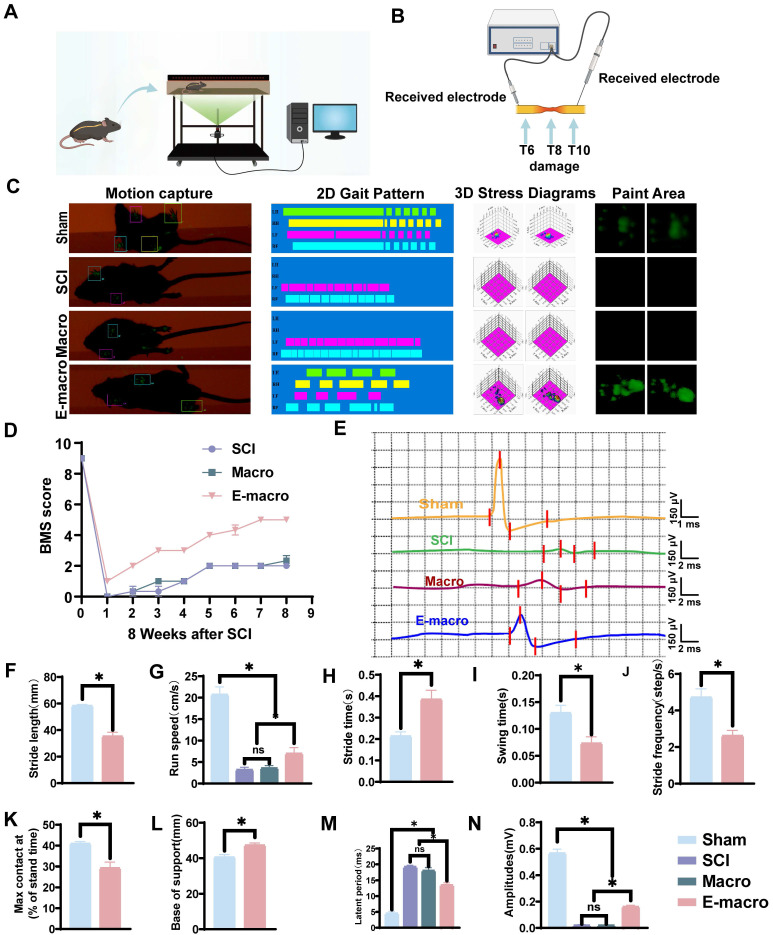
** Engineered macrophages improve motor function and enhance electrophysiological outcomes at 8 weeks after SCI.** (A) Gait analysis of mice at 8 weeks after SCI. (B) Measurement of motor evoked potentials (MEPs). (C) Representative output from Green Walk software in untreated controls, including gait patterns, maximum paw print area, and representative traces of left hind (LH) and right hind (RH) limb movements (LF = left forepaw; RF = right forepaw; LH = left hind paw; RH = right hind paw). (D) BMS locomotor scores. (E) Representative MEP waveforms in different treatment groups. (F) Stride length. (G) Running speed. (H) Gait cycle duration. (I) Swing time. (J) Step frequency. (K) Percentage of the gait cycle during which a single paw maintained maximum ground contact. (L) Support base. (M–N) Quantification of MEP latency and amplitude. Data are presented as mean ± SD; n = 6 biologically independent animals per group for BMS and gait analysis and n = 4 biologically independent animals per group for MEP analysis. Longitudinal BMS scores were analyzed using repeated-measures two-way ANOVA followed by Tukey’s post hoc test. Gait parameters and MEP endpoints were analyzed using one-way ANOVA followed by Tukey’s post hoc test. *p < 0.05; ns, not significant.

At 8 weeks after SCI, locomotor recovery was first assessed using an automated gait analysis system that recorded both two-dimensional and three-dimensional movement parameters (Figure 11A, C). At this time point, mice in the SCI group and the unmodified macrophage-treated group still exhibited severe hindlimb paralysis and were unable to support body weight, such that gait parameters could be obtained only from the forelimbs. In contrast, mice treated with engineered macrophages showed partial recovery of hindlimb function, including detectable ground contact and sustained weight support. The maximum proportion of time spent with a single hind paw in contact with the ground during standing reached 29.23 ± 2.82%, which remained lower than the sham value (40.95 ± 1.01%) but indicated clear recovery of hindlimb weight-bearing ability and stance stability (p < 0.01, Figure 11K). Detailed gait parameters further supported this behavioral recovery. The engineered macrophage-treated group achieved an average stride length of 35.33 ± 3.01 mm, corresponding to approximately 60.53% of the sham value (58.37 ± 0.96 mm), and was significantly greater than that of the SCI and macrophage-treated groups, which were unable to walk (p < 0.01, Figure 11F). Additional gait parameters, including running speed, gait cycle time, swing time, and stride frequency, were also significantly improved in the engineered macrophage-treated group (p < 0.01, Figure 11G–J, L). Together, these data indicate partial restoration of hindlimb locomotor function after engineered macrophage treatment.

This improvement was consistent with the Basso Mouse Scale (BMS) results. At week 8, both the SCI group and the unmodified macrophage-treated group remained at a score of 2, indicating only minimal hindlimb function. By contrast, the engineered macrophage-treated group reached a score of 5, consistent with substantial recovery of autonomous hindlimb movement, although deficits in coordination and overall locomotor performance persisted (Figure 11D).

We then examined whether this behavioral improvement was accompanied by better conduction across the lesion. For motor evoked potential (MEP) recording, the stimulating electrode was placed at T6, two segments rostral to the injury site, and the recording electrode at T10, two segments caudal to the lesion (Figure 11B, E). In sham mice, MEP latency was shortest (4.501 ± 0.67 ms). By contrast, latency was markedly prolonged in both the SCI group and the unmodified macrophage-treated group (18.02 ± 1.00 ms), indicating severe impairment of descending conduction. In the engineered macrophage-treated group, latency was significantly shortened to 13.46 ± 0.76 ms. Although this value did not return to the sham level, it indicates partial recovery of descending conduction after injury (p < 0.01, Figure 11M). MEP amplitude showed the same pattern. The mean amplitude in the engineered macrophage-treated group reached 0.16 ± 0.01 mV, compared with 0.02 ± 0.003 mV in both the SCI and unmodified macrophage-treated groups, corresponding to 7.83-fold and 7.94-fold increases, respectively (p < 0.01, Figure 11N). Although this value remained lower than that of the sham group (0.57 ± 0.03 mV), it still indicated a clear improvement in conduction-related output. Overall, these electrophysiological findings support partial restoration of descending conduction and are in line with the behavioral evidence for incomplete but meaningful recovery of motor function after SCI.

Functionally, the improvements in EMG activity, MEP responses, BMS scores, and gait parameters were consistent with the histological and molecular evidence of reduced inflammation, enhanced neurotrophic signaling, ECM remodeling, axonal repair, remyelination, and synaptic remodeling. This agreement across molecular, histological, electrophysiological, and behavioral readouts suggests that engineered macrophages may promote recovery by both delivering exogenous therapeutic molecules and reshaping endogenous lesion networks. Compared with single-target approaches, this multimodal strategy may better match the multi-stage pathological features of SCI, in which inflammation, matrix disruption, axonal injury, demyelination, and impaired circuit reconnection occur in parallel 69-73.

Several limitations should be acknowledged. First, the present study does not define the relative contribution of ANXA1, GDNF, and CTGF individually. Second, because an M2 macrophage control group was not included, the engineering-specific effects cannot be fully separated from reparative effects related to macrophage phenotype. Third, endogenous microglia, infiltrating macrophages, and intravenously administered engineered cells were not completely distinguished in all *in vivo* analyses. Fourth, the conditioned-medium experiments mainly reflect paracrine effects and do not fully address possible contact-dependent actions of membrane-associated ANXA1. Finally, although *in vitro* ELISA showed sustained short-term secretion after electroporation, the *in vivo* half-life and persistence of the delivered proteins were not directly measured. Future work should refine factor combinations, dosing windows, re-administration strategies, and long-term safety before broader translation is considered.

## Conclusions

In summary, we establish a macrophage-based therapeutic strategy that combines CCR2-associated lesion accumulation with transient and programmable mRNA expression. Engineered CCR2⁺ macrophages were designed to co-deliver ANXA1, GDNF, and CTGF, thereby integrating inflammatory regulation, neurotrophic support, and matrix remodeling within a single cell-based platform. Across molecular, histological, electrophysiological, and behavioral analyses, this strategy was associated with reduced inflammatory activity, enhanced neural and matrix repair, improved remyelination, and partial recovery of motor and conduction-related function after SCI. These findings support engineered macrophages as a flexible and multimodal platform for coordinated microenvironmental regulation and tissue repair after SCI.

## Supplementary Material

Supplementary figures and table, including additional flow cytometric analysis of macrophage phenotype after electroporation, post-electroporation viability and temporal secretion of therapeutic proteins, analysis of the CD45high infiltrating macrophage population in injured spinal cord tissue, morphological quantification of IBA-1 positive inflammatory cells, and gene set enrichment analysis of representative pathways associated with inflammatory regulation, macrophage chemotaxis, extracellular matrix remodeling, and repair-related signaling.

## Data Availability

Data supporting the findings of this study may be obtained from the corresponding author upon reasonable request.

## Abbreviations

ANXA1: annexin A1
BDA: biotinylated dextran amine
BMS: Basso Mouse Scale
BSA: bovine serum albumin
CCL2: C-C motif chemokine ligand 2
CCR2: C-C motif chemokine receptor 2
CD: cluster of differentiation
cDNA: complementary DNA
CTGF: connective tissue growth factor
DAPI: 4′,6-diamidino-2-phenylindole
DEG: differentially expressed gene
DiR: 1,1′-dioctadecyl-3,3,3′,3′-tetramethylindotricarbocyanine iodide
DRG: dorsal root ganglion
ECM: extracellular matrix
EdU: 5-ethynyl-2′-deoxyuridine
eGFP: enhanced green fluorescent protein
ELISA: enzyme-linked immunosorbent assay
EMG: electromyography
FB: Fast Blue
FBS: fetal bovine serum
FN: fibronectin
FPR2: formyl peptide receptor 2
GFAP: glial fibrillary acidic protein
GDNF: glial cell line-derived neurotrophic factor
GFRα-1: GDNF family receptor alpha 1
GO: Gene Ontology
GSEA: gene set enrichment analysis
H&E: hematoxylin and eosin
H₂O₂: hydrogen peroxide
IBA-1: ionized calcium-binding adapter molecule 1
IF: immunofluorescence
IL: interleukin
iNOS: inducible nitric oxide synthase
LN: laminin
MAP2: microtubule-associated protein 2
MBP: myelin basic protein
MEP: motor evoked potential
MPO: myeloperoxidase
mRNA: messenger RNA
NES: normalized enrichment score
NeuN: neuronal nuclei
NF: neurofilament
NG2: neuron-glial antigen 2
PBS: phosphate-buffered saline
PBST: phosphate-buffered saline with Tween 20
PC-12: pheochromocytoma 12
PFA: paraformaldehyde
PI: propidium iodide
qPCR: quantitative polymerase chain reaction
RNA-seq: RNA sequencing
ROS: reactive oxygen species
SCI: spinal cord injury
SD: standard deviation
SDS-PAGE: sodium dodecyl sulfate-polyacrylamide gel electrophoresis
Syn: synaptophysin
TEM: transmission electron microscopy
TGF-β: transforming growth factor beta
TNF-α: tumor necrosis factor alpha
TRIzol: total RNA isolation reagent
Tuj1: class III β-tubulin
TUNEL: terminal deoxynucleotidyl transferase dUTP nick-end labeling
WB: western blot

## References

[1] Ahuja CS, Wilson JR, Nori S, Kotter MRN, Druschel C, Curt A (2017). Traumatic spinal cord injury. Nat Rev Dis Primers.

[2] Zipser CM, Cragg JJ, Guest JD, Fehlings MG, Jutzeler CR, Anderson AJ (2022). Cell-based and stem-cell-based treatments for spinal cord injury: evidence from clinical trials. Lancet Neurol.

[3] Liu S, Sarkar C, Dinizo M, Faden AI, Koh EY, Lipinski MM (2015). Disrupted autophagy after spinal cord injury is associated with ER stress and neuronal cell death. Cell Death Dis.

[4] James SL, Theadom A, Ellenbogen RG, Bannick MS, Montjoy-Venning W, Lucchesi LR (2019). Global, regional, and national burden of traumatic brain injury and spinal cord injury, 1990-2016: a systematic analysis for the Global Burden of Disease Study 2016. Lancet Neurol.

[5] Li Z, Zhao T, Ding J, Gu H, Wang Q, Wang Y (2023). A reactive oxygen species-responsive hydrogel encapsulated with bone marrow derived stem cells promotes repair and regeneration of spinal cord injury. Bioact Mater.

[6] Hu X, Xu W, Ren Y, Wang Z, He X, Huang R (2023). Spinal cord injury: molecular mechanisms and therapeutic interventions. Signal Transduct Target Ther.

[7] Wang Y, Lv HQ, Chao X, Xu WX, Liu Y, Ling GX (2022). Multimodal therapy strategies based on hydrogels for the repair of spinal cord injury. Mil Med Res.

[8] Assinck P, Duncan GJ, Hilton BJ, Plemel JR, Tetzlaff W (2017). Cell transplantation therapy for spinal cord injury. Nat Neurosci.

[9] Olmsted ZT, Stigliano C, Scimemi A, Wolfe T, Cibelli J, Horner PJ (2021). Transplantable human motor networks as a neuron-directed strategy for spinal cord injury. iScience.

[10] Wernig M, Zhao JP, Pruszak J, Hedlund E, Fu D, Soldner F (2008). Neurons derived from reprogrammed fibroblasts functionally integrate into the fetal brain and improve symptoms of rats with Parkinson's disease. Proc Natl Acad Sci U S A.

[11] Wang LL-W, Gao Y, Feng Z, Mooney DJ, Mitragotri S (2024). Designing drug delivery systems for cell therapy. Nat Rev Bioeng.

[12] Yang L, Zhang Y, Zhang Y, Xu Y, Li Y, Xie Z (2022). Live macrophage-delivered doxorubicin-loaded liposomes effectively treat triple-negative breast cancer. ACS Nano.

[13] Xue J, Zhao Z, Zhang L, Xue L, Shen S, Wen Y (2017). Neutrophil-mediated anticancer drug delivery for suppression of postoperative malignant glioma recurrence. Nat Nanotechnol.

[14] Andrzejewska A, Dabrowska S, Lukomska B, Janowski M (2021). Mesenchymal stem cells for neurological disorders. Adv Sci (Weinh).

[15] Lu P, Wang Y, Graham L, McHale K, Gao M, Wu D (2012). Long-distance growth and connectivity of neural stem cells after severe spinal cord injury. Cell.

[16] Tetzlaff W, Okon EB, Karimi-Abdolrezaee S, Hill CE, Sparling JS, Plemel JR (2011). A systematic review of cellular transplantation therapies for spinal cord injury. J Neurotrauma.

[17] Hu XC, Lu YB, Yang YN, Kang XW, Wang YG, Ma B (2021). Progress in clinical trials of cell transplantation for the treatment of spinal cord injury: how many questions remain unanswered?. Neural Regen Res.

[18] Kadoya K, Lu P, Nguyen K, Lee-Kubli C, Kumamaru H, Yao L (2016). Spinal cord reconstitution with homologous neural grafts enables robust corticospinal regeneration. Nat Med.

[19] Ben-David U, Benvenisty N (2011). The tumorigenicity of human embryonic and induced pluripotent stem cells. Nat Rev Cancer.

[20] Wang X, Cao K, Sun X, Chen Y, Duan Z, Sun L (2015). Macrophages in spinal cord injury: phenotypic and functional change from exposure to myelin debris. Glia.

[21] Liu K, Dong X, Wang Y, Wu X, Dai H (2022). Dopamine-modified chitosan hydrogel for spinal cord injury. Carbohydr Polym.

[22] Li Y, Wang Z, Pei S, Chen R, Li Y, Liang Y (2024). Bisphosphonate-based hydrogel with pH-responsive minocycline release inhibits microglia/macrophages of M1 polarization for spinal cord injury therapy. ACS Mater Lett.

[23] Chen R, Zhang H, Pei S, Li Y, Wu Z, He C (2025). Hyaluronan-bisphosphonate conjugate: A macromolecular anti-inflammatory agent and gas delivery system for neuroimmunomodulation in spinal cord injury. Int J Biol Macromol.

[24] Mosser DM, Edwards JP (2008). Exploring the full spectrum of macrophage activation. Nat Rev Immunol.

[25] Gál P, Kravcuková P, Mokrý M, Kluchová D (2009). Chemokines as possible targets in modulation of the secondary damage after acute spinal cord injury: a review. Cell Mol Neurobiol.

[26] Xu P, Zhang F, Chang MM, Zhong C, Sun CH, Zhu HR (2021). Recruitment of γδ T cells to the lesion via the CCL2/CCR2 signaling after spinal cord injury. J Neuroinflammation.

[27] Nunes AK, Rapôso C, de Oliveira WH, Thomé R, Verinaud L, Tovar-Moll F (2016). Phosphodiesterase-5 inhibition promotes remyelination by MCP-1/CCR-2 and MMP-9 regulation in a cuprizone-induced demyelination model. Exp Neurol.

[28] Niemi JP, DeFrancesco-Lisowitz A, Cregg JM, Howarth M, Zigmond RE (2016). Overexpression of the monocyte chemokine CCL2 in dorsal root ganglion neurons causes a conditioning-like increase in neurite outgrowth and does so via a STAT3 dependent mechanism. Exp Neurol.

[29] Shirley JL, de Jong YP, Terhorst C, Herzog RW (2020). Immune Responses to Viral Gene Therapy Vectors. Mol Ther.

[30] Bushman FD (2020). Retroviral Insertional Mutagenesis in Humans: Evidence for Four Genetic Mechanisms Promoting Expansion of Cell Clones. Mol Ther.

[31] Karikó K, Muramatsu H, Welsh FA, Ludwig J, Kato H, Akira S (2008). Incorporation of pseudouridine into mRNA yields superior nonimmunogenic vector with increased translational capacity and biological stability. Mol Ther.

[32] Liu A, Wang X (2022). The pivotal role of chemical modifications in mRNA therapeutics. Front Cell Dev Biol.

[33] Warminski M, Mamot A, Depaix A, Kowalska J, Jemielity J (2023). Chemical modifications of mRNA ends for therapeutic applications. Acc Chem Res.

[34] Walthers CM, Seidlits SK (2015). Gene delivery strategies to promote spinal cord repair. Biomark Insights.

[35] Meyer RA, Neshat SY, Green JJ, Santos JL, Tuesca AD (2022). Targeting strategies for mRNA delivery. Mater Today Adv.

[36] Li X, Qi J, Wang J, Hu W, Zhou W, Wang Y (2024). Nanoparticle technology for mRNA: Delivery strategy, clinical application and developmental landscape. Theranostics.

[37] Yuan M, Han Z, Liang Y, Sun Y, He B, Chen W (2023). mRNA nanodelivery systems: targeting strategies and administration routes. Biomater Res.

[38] Crowley ST, Fukushima Y, Uchida S, Kataoka K, Itaka K (2019). Enhancement of motor function recovery after spinal cord injury in mice by delivery of brain-derived neurotrophic factor mRNA. Mol Ther Nucleic Acids.

[39] Qin S, Tang X, Chen Y, Chen K, Fan N, Xiao W (2022). mRNA-based therapeutics: powerful and versatile tools to combat diseases. Signal Transduct Target Ther.

[40] Squair JW, Tigchelaar S, Moon KM, Liu J, Tetzlaff W, Kwon BK (2018). Integrated systems analysis reveals conserved gene networks underlying response to spinal cord injury. Elife.

[41] McArthur S, Cristante E, Paterno M, Christian H, Roncaroli F, Gillies GE (2010). Annexin A1: a central player in the anti-inflammatory and neuroprotective role of microglia. J Immunol.

[42] Novizio N, Belvedere R, Pessolano E, Morello S, Tosco A, Campiglia P (2021). ANXA1 contained in EVs regulates macrophage polarization in tumor microenvironment and promotes pancreatic cancer progression and metastasis. Int J Mol Sci.

[43] Yao X, Sun C, Fan B, Zhao C, Zhang Y, Duan H (2021). Neurotropin exerts neuroprotective effects after spinal cord injury by inhibiting apoptosis and modulating cytokines. J Orthop Translat.

[44] Cintrón-Colón AF, Almeida-Alves G, Boynton AM, Spitsbergen JM (2020). GDNF synthesis, signaling, and retrograde transport in motor neurons. Cell Tissue Res.

[45] Zou Z, Liu R, Wang Y, Tan H, An G, Zhang B (2023). Protein arginine methyltransferase 8 regulates ferroptosis and macrophage polarization in spinal cord injury via glial cell-derived neurotrophic factor. CNS Neurosci Ther.

[46] Walker MJ, Xu X-M (2018). History of glial cell line-derived neurotrophic factor (GDNF) and its use for spinal cord injury repair. Brain Sciences.

[47] Wang Y, Kong QJ, Sun JC, Yang Y, Wang HB, Zhang Q (2018). Lentivirus-mediated silencing of the CTGF gene suppresses the formation of glial scar tissue in a rat model of spinal cord injury. Spine J.

[48] Fu M, Peng D, Lan T, Wei Y, Wei X (2022). Multifunctional regulatory protein connective tissue growth factor (CTGF): A potential therapeutic target for diverse diseases. Acta Pharm Sin B.

[49] Mokalled MH, Patra C, Dickson AL, Endo T, Stainier DY, Poss KD (2016). Injury-induced ctgfa directs glial bridging and spinal cord regeneration in zebrafish. Science.

[50] Rios FJ, Touyz RM, Montezano AC (2017). Isolation and differentiation of murine macrophages. Methods Mol Biol.

[51] Lu B, Jia S, Wang Z, Wu W, Yan L, Zhu L (2023). Sensory-motor coupling electrical stimulation driven by a bionic Z-structured triboelectric nanogenerator improves functional recovery from spinal cord injury. Nano Energy.

[52] Zhu L, Jia S, Liu T, Yan L, Huang D, Wang Z (2020). Aligned PCL fiber conduits immobilized with nerve growth factor gradients enhance and direct sciatic nerve regeneration. Adv Funct Mater.

[53] Chu T, Wang Z, Pe’er D, Danko CG (2022). Cell type and gene expression deconvolution with BayesPrism enables Bayesian integrative analysis across bulk and single-cell RNA sequencing in oncology. Nat Cancer.

[54] Li C, Wu Z, Zhou L, Shao J, Hu X, Xu W (2022). Temporal and spatial cellular and molecular pathological alterations with single-cell resolution in the adult spinal cord after injury. Signal Transduct Target Ther.

[55] Wei J, Chen P, Gupta P, Ott M, Zamler D, Kassab C (2020). Immune biology of glioma-associated macrophages and microglia: functional and therapeutic implications. Neuro Oncol.

[56] Liu NK, Zhang YP, Han S, Pei J, Xu LY, Lu PH (2007). Annexin A1 reduces inflammatory reaction and tissue damage through inhibition of phospholipase A2 activation in adult rats following spinal cord injury. J Neuropathol Exp Neurol.

[57] Zheng J, Chen T, Wang K, Peng C, Zhao M, Xie Q (2024). Engineered multifunctional zinc-organic framework-based aggregation-induced emission nanozyme for accelerating spinal cord injury recovery. ACS Nano.

[58] Prinz M, Jung S, Priller J (2019). Microglia biology: one century of evolving concepts. Cell.

[59] Tang Y, Liu J, Wang Y, Yang L, Han B, Zhang Y (2021). PARP14 inhibits microglial activation via LPAR5 to promote post-stroke functional recovery. Autophagy.

[60] Zhou H, Yan L, Huang H, Li X, Xia Q, Zheng L (2023). Tat-NTS peptide protects neurons against cerebral ischemia-reperfusion injury via ANXA1 sumoylation in microglia. Theranostics.

[61] Li Y, Liang Y, He C, Yao R, Jian K, Shi L (2025). A dual-drug sequential delivery hydrogel for programmatic microglia/macrophage polarization and function recovery in spinal cord injury. Mater Today Bio.

[62] Li X, Pan J, Li H, Li G, Liu X, Liu B (2020). DsbA-L mediated renal tubulointerstitial fibrosis in UUO mice. Nat Commun.

[63] Li H, Ghorbani S, Zhang R, Ebacher V, Stephenson EL, Keough MB (2023). Prominent elevation of extracellular matrix molecules in intracerebral hemorrhage. Front Mol Neurosci.

[64] Fawcett JW, Oohashi T, Pizzorusso T (2019). The roles of perineuronal nets and the perinodal extracellular matrix in neuronal function. Nat Rev Neurosci.

[65] Tran AP, Warren PM, Silver J (2018). The Biology of Regeneration Failure and Success After Spinal Cord Injury. Physiol Rev.

[66] Xiao S, Zhang Y, Liu Z, Li A, Tong W, Xiong X (2023). Alpinetin inhibits neuroinflammation and neuronal apoptosis via targeting the JAK2/STAT3 signaling pathway in spinal cord injury. CNS Neurosci Ther.

[67] Francos-Quijorna I, Sánchez-Petidier M, Burnside ER, Badea SR, Torres-Espin A, Marshall L (2022). Chondroitin sulfate proteoglycans prevent immune cell phenotypic conversion and inflammation resolution via TLR4 in rodent models of spinal cord injury. Nat Commun.

[68] Liu Y, Hu C, He S, Liu R, Zhao Y, Wang Y (2026). Biomimetic core-shell GelMA microspheres co-delivering ANXA1, NGF, and fibronectin enable phase-matched immunomodulation and neurorepair after spinal cord injury. Theranostics.

[69] Zheng B, Tuszynski MH (2023). Regulation of axonal regeneration after mammalian spinal cord injury. Nat Rev Mol Cell Biol.

[70] Zuo Y, Ye J, Cai W, Guo B, Chen X, Lin L (2023). Controlled delivery of a neurotransmitter-agonist conjugate for functional recovery after severe spinal cord injury. Nat Nanotechnol.

[71] Anderson MA, O’Shea TM, Burda JE, Ao Y, Barlatey SL, Bernstein AM (2018). Required growth facilitators propel axon regeneration across complete spinal cord injury. Nature.

[72] Sofroniew MV (2018). Dissecting spinal cord regeneration. Nature.

[73] Courtine G, Sofroniew MV (2019). Spinal cord repair: advances in biology and technology. Nat Med.

