# Supplementary material for: Engineered CCR2 positive macrophages coordinate immunoregulation with neural regeneration and matrix remodeling after spinal cord injury
Source: Theranostics. 2026 Jun 10;16(13):7388–418. doi: 10.7150/thno.134560 (PMC13295169; doi:10.7150/thno.134560)
Supplement: Supplementary file 1 — Supplementary figures and table, including additional flow cytometric analysis of macrophage phenotype after electroporation, post-electroporation viability and temporal secretion of therapeutic proteins, analysis of the CD45high infiltrating macrophage population in injured spinal cord tissue, morphological quantification of IBA-1 positive inflammatory cells, and gene set enrichment analysis of representative pathways associated with inflammatory regulation, macrophage chemotaxis, extracellular matrix remodeling, and repair-related signaling. [file thnov16p7388s1.pdf]

Supplementary materials

Engineered CCR2 positive macrophages coordinate immunoregulation with neural regeneration and matrix remodeling after spinal cord injury

Yuqi Zhao[1,2*], Tao Xie[1,2*], Yanming Ma[1,2*], Yuhao Wang[1,2], Shenghang Liu[1,2], Hui Li[1,2], Youjun Liu[1,2], Renfeng Liu[1,2], Hailiang Xu[1,2], Cheng Ju[1,2], Weidong Wu[1,2], Yifan Wang[1,2], Siyuan He[1,2], Rongjin Luo[1,2], Dageng Huang[1,2], Shuaijun Jia[1,2], Chunping Hu[1,2], Liang Yan [1,2]✉, Zhiyuan Wang[1,2]✉, Lei Zhu[1,2]✉

1 Department of Spine Surgery, Honghui Hospital, Xi'an Jiaotong University, Xi'an, Shaanxi 710054, China

2 Shaanxi Key Laboratory of Spine Bionic Treatment, Xi’an, Shaanxi 710054, China

*Yuqi Zhao, Tao Xie, Yanming Ma contributed equally to this work

✉Corresponding authors. Liang Yan, Zhiyuan Wang and Lei Zhu.

E-mail: yanliangdr5583@163.com (for Pro. Liang Yan) ; wangzy199561@stu.xjtu.edu.cn (for Ph.D. Zhiyuan Wang); zhulei619@xjtu.edu.cn (for Pro. Lei Zhu)

Supporting Information

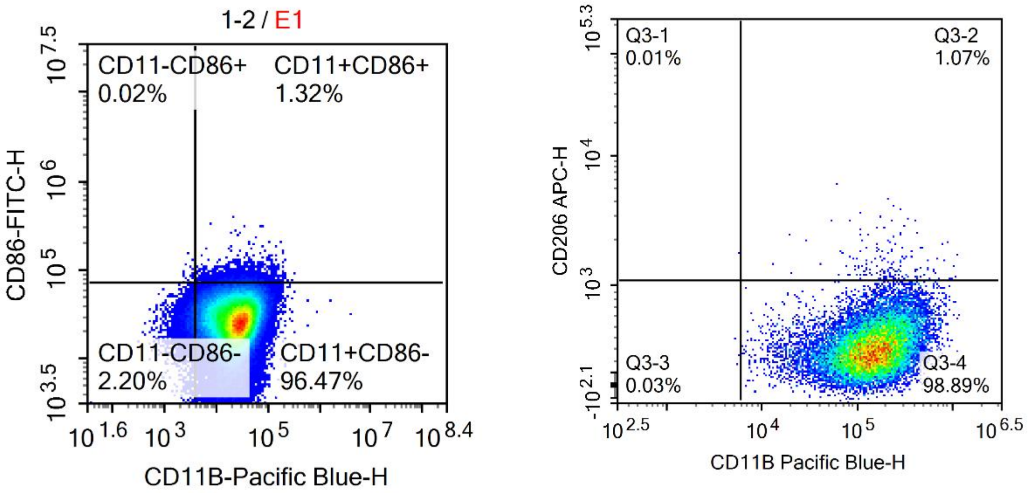

Figure S1. Flow cytometric analysis of macrophage phenotype after electroporation.

Representative pseudocolor dot plots showing the expression of CD86 and CD206 in $CD11b^+$ cells. The left panel shows the proportion of $CD11b^+CD86^+$ cells, and the right panel shows the proportion of $CD11b^+CD206^+$ cells. Percentages of cells in each quadrant are indicated in the plots.

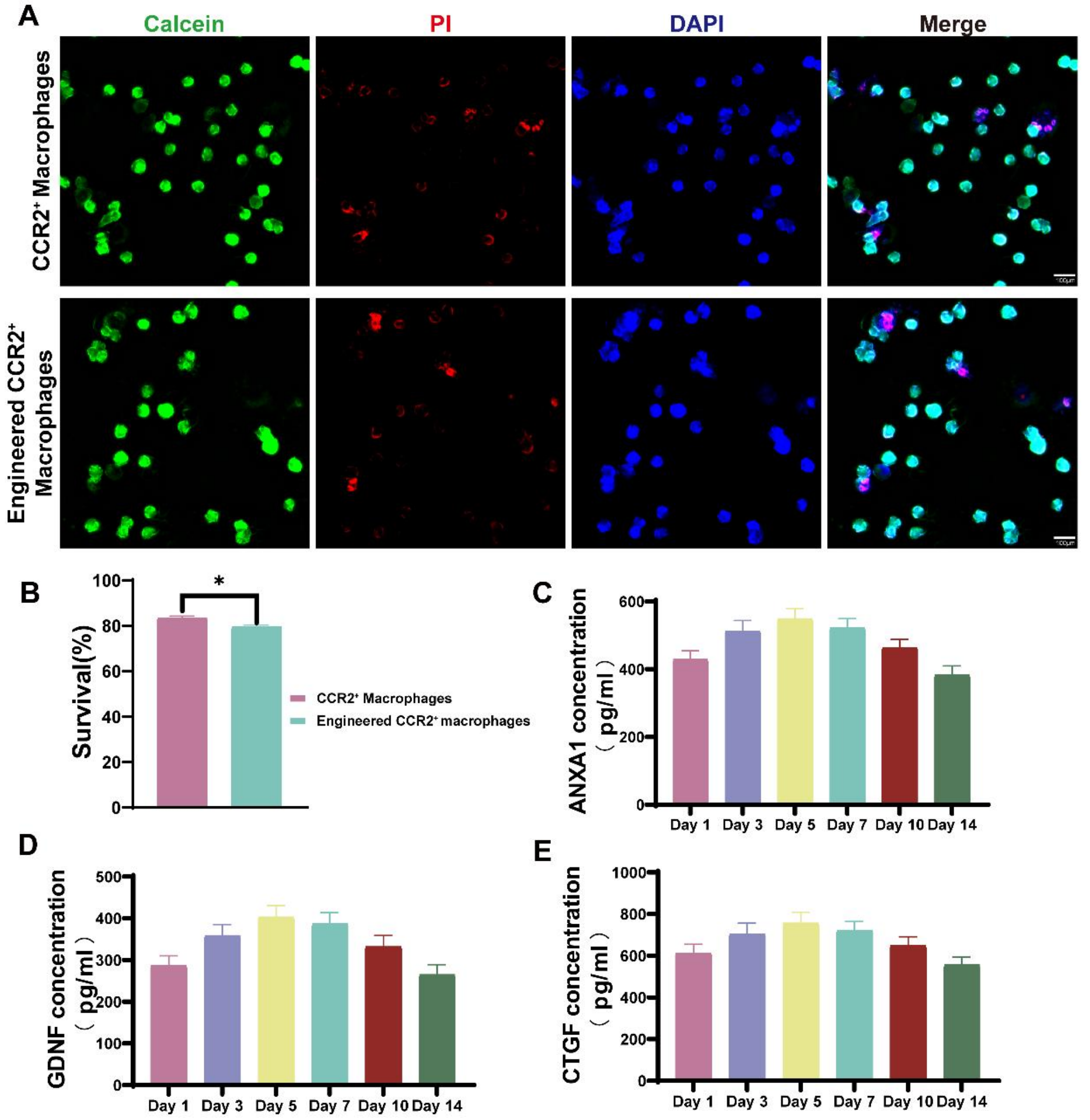

Figure S2. Post-electroporation viability and temporal secretion of therapeutic proteins in engineered CCR2$^+$ macrophages.

(A) Representative Calcein-AM/PI staining images of CCR2$^+$ macrophages and engineered CCR2$^+$ macrophages after electroporation. Live cells are shown in green (Calcein-AM), dead cells in red (PI), and nuclei in blue (DAPI). (B) Quantification of post-electroporation cell survival in CCR2$^+$ macrophages and engineered CCR2$^+$ macrophages. (C–E) ELISA-based quantification of secreted ANXA1 (C), GDNF (D),

and CTGF (E) in culture supernatants collected from engineered macrophages on days 1, 3, 5, 7, 10, and 14 after electroporation. Data are presented as mean ± SD. $*p < 0.05$.

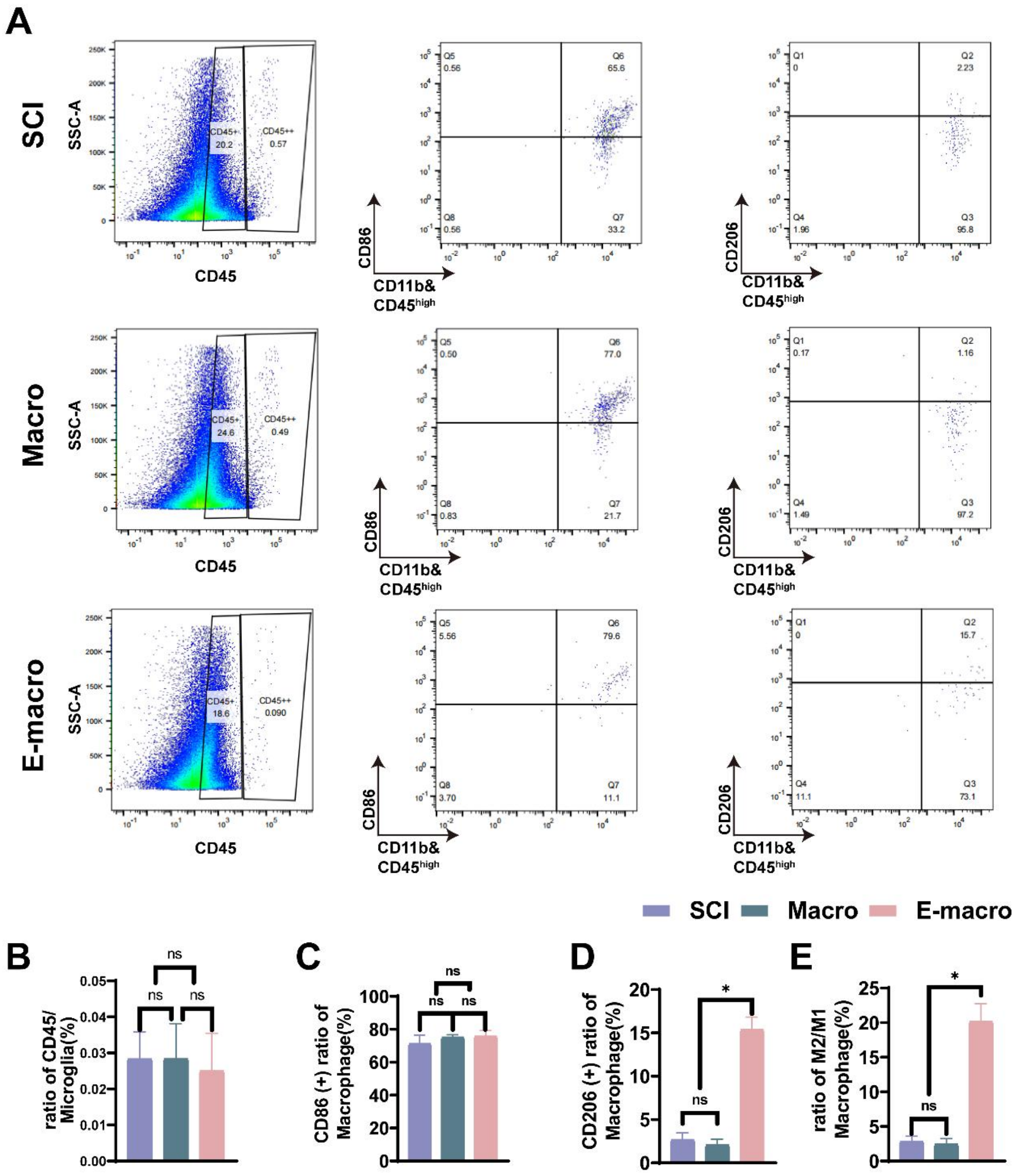

Figure S3. Flow cytometric analysis of the minor CD45^{high} infiltrating macrophage population in the injured spinal cord at day 7 after SCI.

(A) Representative gating strategy showing separation of CD45^{low} and CD45^{high} myeloid populations, followed by analysis of CD86 and CD206 expression within the CD11b^{+} CD45^{high} infiltrating macrophage gate in the SCI, macrophage-treated (Macro), and engineered macrophage-treated (E-macro) groups.(B) Quantification of the proportion of CD45^{high} infiltrating macrophages among total myeloid cells.(C) Quantification of the percentage of CD86^{+} macrophages within the CD45^{high} infiltrating macrophage population.(D) Quantification of the percentage of CD206^{+} macrophages within the CD45^{high} infiltrating macrophage population.(E) Quantification of the CD206/CD86 ratio in the CD45^{high} infiltrating macrophage population.

Data are presented as mean ± SD. *$p < 0.05$; ns, not significant.

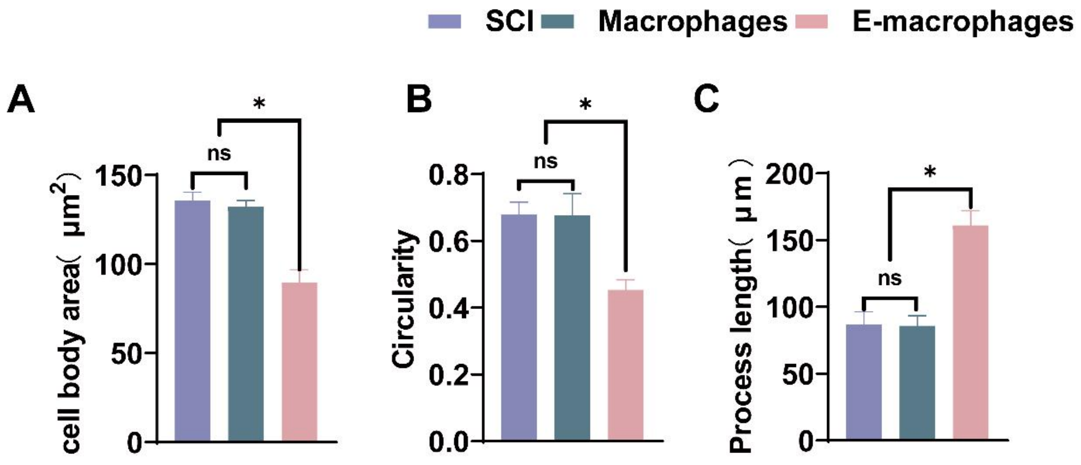

Figure S4. Morphological quantification of IBA-1^{+} inflammatory cells in the injured spinal cord.

(A) Quantification of cell body area of IBA-1⁺ inflammatory cells in the SCI, macrophage-treated, and engineered macrophage-treated groups. (B) Quantification of circularity of IBA-1⁺ inflammatory cells across groups. (C) Quantification of total process length of IBA-1⁺ inflammatory cells across groups. Data are presented as mean ± SD. *$p < 0.05$; ns, not significant.

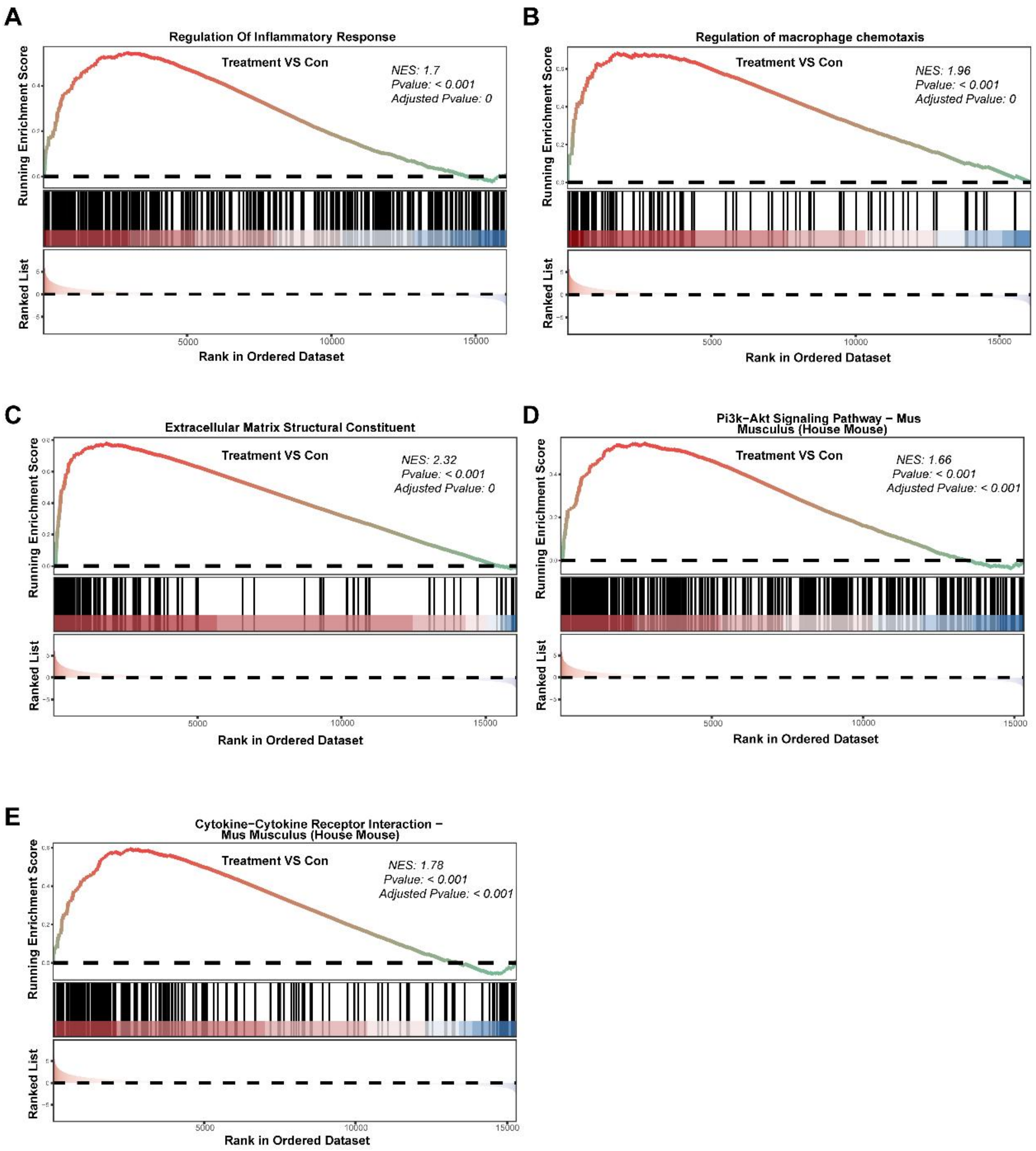

Figure S5. Gene set enrichment analysis of representative pathways associated with inflammatory regulation, macrophage chemotaxis, extracellular matrix remodeling, and repair-related signaling in chronic SCI tissue.

(A) GSEA plot showing enrichment of the regulation of inflammatory response gene set in the engineered macrophage treatment group relative to the control group. (B) GSEA plot showing enrichment of the regulation of macrophage chemotaxis gene set in the engineered macrophage treatment group relative to the control group. (C) GSEA plot showing enrichment of the extracellular matrix structural constituent gene set in the engineered macrophage treatment group relative to the control group. (D) GSEA plot showing enrichment of the PI3K–Akt signaling pathway in the engineered macrophage treatment group relative to the control group. (E) GSEA plot showing enrichment of the cytokine–cytokine receptor interaction pathway in the engineered macrophage treatment group relative to the control group. Normalized enrichment score (NES), nominal P value, and adjusted P value are indicated in each panel. Con, control group.

**Gene**	**Forward primer 5′–3′**	**Reverse primer 5′–3′**
Ccl2	TTAAAAACCTGGATCGGAACCAA	GCATTAGCTTCAGATTTACGGGT
Fgf2	AAGCGGCTCTACTGCAAGAAC	GGTCCCGTTTTGGATCCGAG

Igf1	GCTCTTCAGTTCGTGTGTGG	GCCTCCTTAGATCACAGCTCC
Ntn4	GAGGAGGAGCAGACCATCAA	CTCCTTGGTGATGTTGGTGA
Gdnf	GACTCCAATATGCCCGAAGATT	CTTGTCACTCACCAGCCTTCT
Anxa1	GACACCAAGGGTGTGATGGT	CAGTCTGTTGGCAGCTTCCT
Fpr2	CCAGGACTTTCGGTTCCTGT	AGTCCAGGCGTTTCTGTTGT
Cd86	TGTTTCCGTGGAGACGCAAG	TTGAGCCTTTGTAAATGGGCA
Col1a1	GCTCCTCTTAGGGGCCACT	CCACGTCTCACCATTGGGG
Ctgf	GGGCCTCTTCTGCGATTTC	ATCCAGGCAAGTGCATTGGTA
Lama2	CAGTGCTGCTGATGACCAAC	TGCAGGTAGTTGGTGATGGT
Gfap	AGAAAACCGCATCACCATTC	TCACATCACCACGTCCTTGT
Plp1	ACTACAAGACCACCATCTGCC	GGCAATCATGAAGGTGAGGA
Atp7a	TCTGCTGTTGTGGTTGGTGT	AGGAGGTCATCAGGCTGTTC
Gapdh	CATCACTGCCACCCAGAAGACTG	ATGCCAGTGAGCTTCCCGTTCAG

Table S1. Primer sequences used for qPCR analysis.

All primers are listed in the 5′–3′ direction. *Gapdh* was used as the internal reference gene. Primer specificity should be confirmed by melting-curve analysis and agarose gel electrophoresis or Primer-BLAST validation.
